# DNA methylation and hydroxymethylation characterize the identity of D1 and D2 striatal projection neurons

**DOI:** 10.1038/s42003-022-04269-w

**Published:** 2022-12-01

**Authors:** Lucile Marion-Poll, Jean-Pierre Roussarie, Lieng Taing, Cloelia Dard-Dascot, Nicolas Servant, Yan Jaszczyszyn, Emmanuelle Jordi, Eskeatnaf Mulugeta, Denis Hervé, Déborah Bourc’his, Paul Greengard, Claude Thermes, Jean-Antoine Girault

**Affiliations:** 1 INSERM UMR-S1270, Paris, 75005 France; 2 Sorbonne Université, Faculty of Sciences and Engineering, Paris, 75005 France; 3 Institut du Fer à Moulin, Paris, 75005 France; 4 Institut Curie, PSL Research University, CNRS UMR3215, INSERM U934, Paris, 75005 France; 5 Laboratory of Molecular and Cellular Neuroscience, The Rockefeller University, New York, NY 10065 USA; 6 Université Paris-Saclay, CEA, CNRS, Institute for Integrative Biology of the Cell (I2BC), Gif-sur-Yvette, 91198 France; 7 Institut Curie, INSERM U900, CBIO-Centre for Computational Biology, Mines Paris Tech, PSL-Research University, Paris, 75005 France; 8 Present Address: Department of Basic Neurosciences, Faculty of Medicine, University of Geneva, Geneva, 1211 Switzerland; 9 Present Address: Department of Anatomy & Neurobiology, Boston University Chobanian & Avedisian School of Medicine, Boston, MA 02118 USA; 10 Present Address: UMR1166 Inserm and Sorbonne Université, Faculty of Medicine, Paris, 75013 France; 11 Present Address: Coave Therapeutics, Paris, 75014 France; 12 Present Address: Erasmus University Medical Center (Erasmus MC), Department of Cell Biology, Rotterdam, 3000 CA The Netherlands

**Keywords:** Epigenetics in the nervous system, Epigenetics

## Abstract

Neuronal DNA modifications differ from those in other cells, including methylation outside CpG context and abundant 5-hydroxymethylation whose relevance for neuronal identities are unclear. Striatal projection neurons expressing D1 or D2 dopamine receptors allow addressing this question, as they share many characteristics but differ in their gene expression profiles, connections, and functional roles. We compare translating mRNAs and DNA modifications in these two populations. DNA methylation differences occur predominantly in large genomic clusters including differentially expressed genes, potentially important for D1 and D2 neurons. Decreased gene body methylation is associated with higher gene expression. Hydroxymethylation differences are more scattered and affect transcription factor binding sites, which can influence gene expression. We also find a strong genome-wide hydroxymethylation asymmetry between the two DNA strands, particularly pronounced at expressed genes and retrotransposons. These results identify novel properties of neuronal DNA modifications and unveil epigenetic characteristics of striatal projection neurons heterogeneity.

## Abstract (web summary)

Striatal projection neurons expressing the D1 or D2 dopamine receptors are probed for translating mRNAs and DNA modifications, identifying differential methylation signatures between the cell types and genome-wide hydroxymethylation asymmetry between the two DNA strands.

## Introduction

Epigenetic marks, including DNA modifications, play a key role in defining cell identities. An intriguing area concerns the role of epigenetic modifications in controlling specific properties of neurons which are long-lived post-mitotic cells, dynamically regulated by external stimuli, including environment and life experiences. In mammalian cells, cytosines can be methylated at the fifth carbon, generally when followed by a guanine (CG), a context where both strands can be symmetrically methylated^1^, whereas in neurons, up to half of the DNA methylation (5mC) is found in a non-CG (i.e., CH) context^2^. 5mC can be oxidized to 5-hydroxymethylated-cytosine by the TET (ten-eleven translocation) enzymes, and this modification is especially abundant in neurons^3^. DNA hydroxymethylation (5hmC) is an intermediate for demethylation during development^4,5^ and may play specific roles in the adult brain, through the recruitment of 5hmC-specific binding proteins^6^.

DNA modification in neurons contributes to neuronal development and function. Mutations in DNA methyltransferase or chromatin modifier genes are associated with brain developmental disorders^7,8^. The methylcytosine binding protein 2 (MECP2), which binds to 5mC and 5hmC, is particularly abundant in neurons^9,10^ and is mutated in Rett syndrome, the second cause of mental disability in girls. 5mC and 5hmC can be modified following stimulation^11^ and may contribute to the formation and stabilization of long-term memory^12^.

The striatum has essential roles in movement control, action selection, and reinforcement learning. This is achieved through the existence of two main types of GABAergic striatal projection neurons (SPNs, a.k.a. medium-size spiny neurons or MSNs), which have opposite but complementary roles^13,14^. SPNs that project to the substantia nigra pars reticulata form the direct striatonigral pathway and express dopamine D1 receptors, while SPNs that project to the external globus pallidus participate in the indirect pathway and express D2 receptors^15^, with very few neurons expressing both types of receptors^16^. D1 receptors are activated by phasic increases in extracellular levels of dopamine, triggered by unexpected rewards^17^ and other salient stimuli^18^. D1- and D2-SPNs have opposite effects on locomotion^19^ but shape behavior in an integrated manner^20^. These neurons are involved in major pathological conditions, including Parkinson’s disease and addiction^21,22^. Histone and DNA modifications in the striatum are altered by the treatment of Parkinson’s disease, levodopa^23^, and by drugs of abuse, while epigenetic modifiers or epigenetic editing can affect addictive behaviors^24^.

D1- and D2-SPNs are very closely related^25^, but differentially express several hundreds of genes^16,26^. During development, both populations originate from the lateral ganglionic eminence and migrate to the future striatum, where they are intermingled. The generation of SPNs starts around E10.5 in mice and continues until birth^27^. The precise mechanisms determining their differential projections are not known, but key transcription factors (TFs) for their identities have been identified, including *Ebf1*^28^ or *Isl1*^29^ for D1-SPNs and *Sp9*^30^ or *Six3*^31^ for D2-SPNs. The expression of specific markers, notably D1- and D2-receptors, starts early on but keeps increasing after birth^32,33^. Previous studies have explored the genome-wide distribution of DNA modifications in very different neuronal types, or in mixed neurons compared to other cell types^2,9,34–38^. In contrast, comparing D1- and D2-SPNs provides an excellent model to explore the specificities of DNA modification established during terminal neuronal differentiation. Here, we characterize the methylomes and hydroxymethylomes of the two types of SPNs in relation to their translatome. Our aim was to investigate D1/D2 differentially modified regions and to compare them with gene expression differences rather than establishing quantitative profiles of these DNA modifications in each cell type at a single base resolution. We, therefore, chose enrichment-based methods over bisulfite-based methods for this study, because they allow direct readouts of 5mC and 5hmC, and have a good sensitivity for sparse modifications such as 5hmC^39^. Antibody-based approaches can show some biases^40^, but these would be identical between SPNs.

We find that most 5mC differences map to 15 structural domains of hundreds of kilobases, which include D1/D2-specific genes, likely to be of key importance for SPNs specificities. We show at the single gene level that decreased 5mC at promoters or gene bodies is correlated with increased expression of differentially expressed genes. 5hmC differences preferentially affect transcription factor binding sites (TFBSs) and are also associated with differential gene expression. We find strand asymmetry of both DNA modifications, but much more pronounced for 5hmC. Hydroxymethylation is asymmetric all over the genome, with nested asymmetric regions with a bimodal size distribution of up to 1 Mb. Asymmetry is high in retrotransposons and highly transcribed genes, which depends on the direction of transcription. Our study highlights specific features of neuronal DNA modifications in relation to gene expression and characterizes their differences between the two main populations of striatal dopamine-sensitive neurons.

## Results

### Translating mRNA and DNA modifications patterns in D1 and D2 neurons

**Fig. 1:**
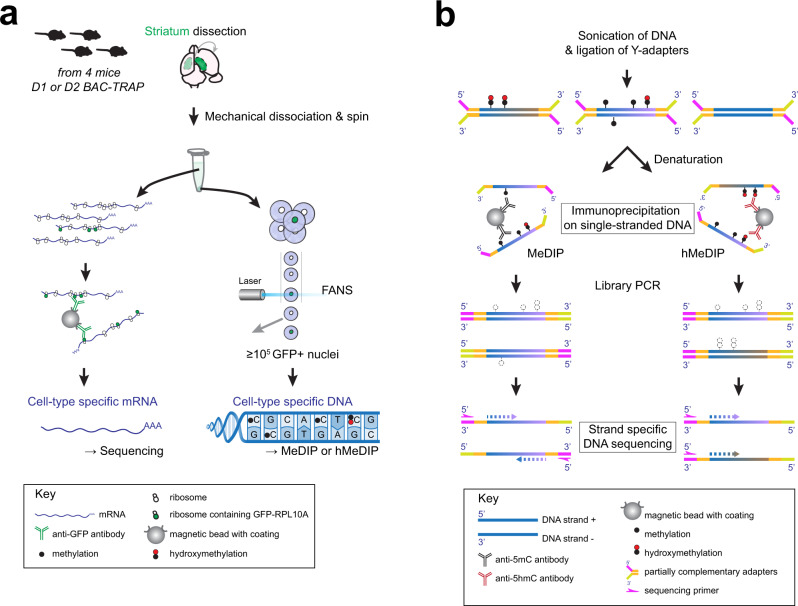
Experimental strategy. **a** Cell population-specific mRNA and DNA preparation from D1-BAC-TRAP and D2-BAC-TRAP mice. D1- or D2-specific mRNA was recovered by immunoprecipitation of ribosomes containing EGFP-RPL10A with anti-GFP antibodies. D1- or D2-specific DNA was obtained by FANS using the fluorescence of nucleoli containing EGFP-RPL10A during ribosomal assembly, followed by DNA extraction. **b** Strand-specificity of the MeDIP-seq and hMeDIP-seq procedures.

To assess epigenomic and transcriptomic patterns in D1 and D2 neurons, we used mice carrying a bacterial artificial chromosome (BAC) that express the ribosomal protein L10a (RPL10A) fused to EGFP under the control of either *Drd1* (D1R) or *Drd2* (D2R) promoters^25,26^ (Supplementary Fig. 1a). These BAC-TRAP (translating ribosome affinity purification) mice allow immunopurification and sequencing of cell-type specific translating mRNAs (TRAP-seq). As RPL10A is also abundant in the nucleoli where ribosomes are assembled (Supplementary Fig. 1b), the GFP-positive nuclei from these mice can be sorted by fluorescence-activated nuclear sorting^3^ (FANS) with very high purity (>97%, see Supplementary Fig. 2a and Methods for the gating strategy). Because the nuclear tagging is not part of the chromatin, it avoids the critical pitfall of disturbing chromatin organization and epigenomic profiles^41^. We combined D1/D2-TRAP-Seq with 5mC- and 5hmC-immunoprecipitation (MeDIP and hMeDIP, respectively) followed by DNA strand-specific sequencing (Fig. 1a, b, and Methods).

**Fig. 2:**
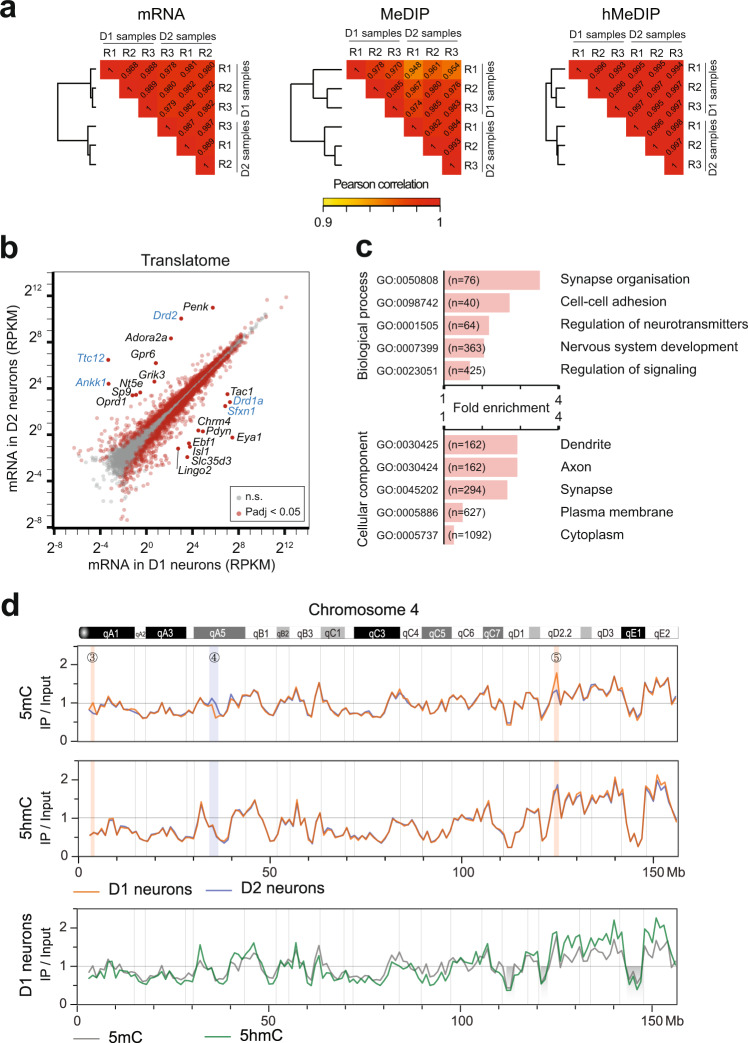
Similarities and differences of striatal D1 and D2 translatomes, methylomes, and hydroxymethylomes. **a** Hierarchical clustering and Pearson correlations between D1 and D2 samples. Rn, replicate #n. **b** Scatter plot of gene expression levels in D1 and D2 neurons. The most different gene names are indicated, with those included in the BAC in blue (excluded from further analysis). **c** Enriched gene ontology terms (FDR < 10^−4^ for biological processes and FDR < 10^−21^ for cellular components) identified with the differentially expressed genes (*n* = 1963). **d** MeDIP enrichment of 5mC and hMeDIP 5hmC (expressed as a number of reads in IP/number of reads in input per 1 megabase bin) throughout chromosome 4 (shown as an example), for D1 and D2 neurons. The blue and orange vertical shaded areas highlight the main D1/D2 differences in 5mC (numbering corresponds to Fig. 3a). Previously described 5mC deserts^2^ are shaded gray. The chromosome regions and bands are indicated at the top.

The results were highly reproducible between the three replicates (each comprised of bilateral striata from 4 mice), and the two neuronal types showed very close proximity (Fig. 2a). The TRAP-seq was clearly enriched for striatal neuronal markers and contained very low levels of markers from other cell types (as identified by Gokce et al.^16^, Supplementary Fig. 2b) notably cholinergic interneurons (*Chat*, *Scl18a3*, Supplementary Table 1). In addition, it showed very high specificity in all samples, as illustrated by the expression of *Drd1* and *Drd2* genes (Supplementary Fig. 2c). Genes known to be specifically expressed in D1 (e.g., *Pdyn*, *Tac1*, and *Slc35d3*) or D2 neurons (e.g., *Adora2a* and *Penk*)^26,42^ were, as expected, highly differentially expressed), along with 1963 other genes for an adjusted Pvalue (Padj) of 0.05 (Fig. 2b, Supplementary Fig. 2d, Supplementary Table 1), mostly related to neuronal functions (Fig. 2c). Our results were in accordance with previous results obtained with various methods^16,26,42–44^ (Supplementary Fig. 2e). The DNA methyltransferases (DNMT) genes were not differentially expressed, while two of the TET enzymes showed a higher expression in D2-SPNs (*Tet1*, +39%, Padj = 1.7 × 10^−2^, and *Tet2*, +44%, Padj = 3.8 × 10^−2^, Supplementary Table 1).

The methylomes and hydroxymethylomes were remarkably similar between D1- and D2-SPNs (Fig. 2d), as expected because of their close similarity. As 5hmC is an intermediate for demethylation, the two profiles showed an overall similarity. However, we observed some large regions already visible at the megabase scale, where D1 and D2 5mC, but not 5hmC, appeared different (highlighted in Fig. 2d). We, therefore, analyzed how the D1/D2 differences in DNA modification are organized at a finer scale.

### 5mC and 5hmC form cell type-specific clusters

**Fig. 3:**
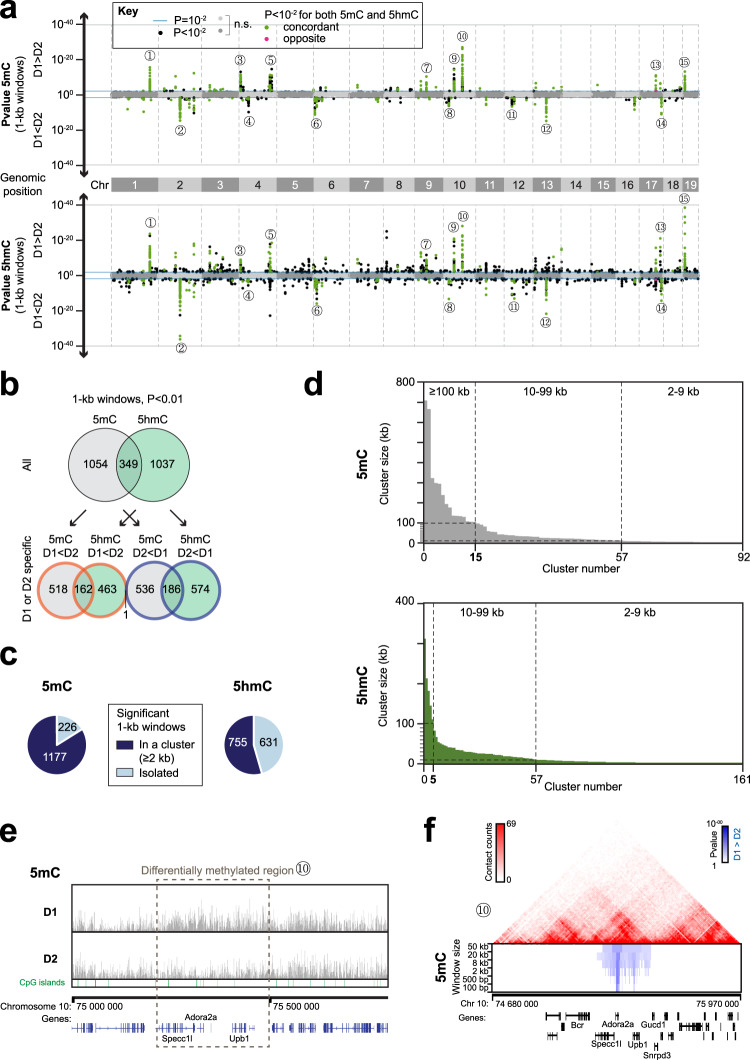
Large clusters of D1/D2 5mC differences and more scattered 5hmC differences. **a** Manhattan plots of 5mC and 5hmC differences along the autosomes. Dots represent 1-kb windows. The *P* value is plotted upwards when the modification is enriched in D1 vs. D2 neurons, and downwards otherwise. Circled numbers represent the biggest clusters of windows, numbered from left to right, which are referred to in the text and in other panels of this figure. *P*, adjusted *P* value. **b** Venn diagrams of the significant 1-kb windows. **c** Clustering of the significant 1-kb windows. The pie charts show the proportions of 1-kb windows that are in a cluster (as defined in Methods). **d** Distribution of the cluster sizes for 5mC and 5hmC. Clusters are ordered from the largest to the smallest. **e** Genome browser representation of the differentially methylated cluster number 10 (as numbered in panel (**a**)). **f** Hi–C interaction frequencies from the study of Bonev et al.^45^ displayed as a two-dimensional heatmap, superimposed with differentially methylated regions of cluster 10. The differentially methylated regions were analyzed with multiple window sizes as indicated (see Methods).

To further analyze differentially methylated or hydroxymethylated regions between D1 and D2 cells, we first compared them using adjacent 1-kb windows over the genome (Fig. 3a), excluding the BAC transgene sequences. We identified 1403 differentially methylated and 1386 differentially hydroxymethylated 1-kb windows (Supplementary Tables 2 and 3). The majority of the differential 5mC windows were grouped in specific regions of the genome, which were already visible at the megabase scale (Figs. 3a,  2d). A quarter of them was also differentially hydroxymethylated, in a concordant manner (i.e., more hydroxymethylated and more methylated in the same cell type, Fig. 3b). To estimate the size of the differentially modified regions, we clustered significantly differentially modified 1-kb windows which were in close proximity (see Methods for details). Among 1403 differentially methylated 1-kb windows, 84% could be grouped (Fig. 3c) into 92 regions of 2 kb or more (Fig. 3d, Supplementary Table 4). We found 15 large clusters >100 kb, as illustrated in Fig. 3e for cluster #10, which spans 237 kb and includes *Adora2a* and other genes (Supplementary Table 5). All the main 5mC clusters also contained some 5hmC differences (Fig. 3a). However, only 55% of the differentially hydroxymethylated 1-kb windows could be grouped (Fig. 3c), into 161 clusters (Fig. 3d, Supplementary Table 6), including five large clusters >100 kb (Fig. 3d, Supplementary Table 7), four of which overlapped large 5mC clusters. Thus, the D1/D2 5mC differences were more clustered across the genome than the 5hmC differences, which tended to be more scattered. We, therefore, investigated whether the clusters of 5mC differences correspond to chromatin regions relevant for D1 and D2 identities.

### D1/D2 5mC clusters contain differentially expressed genes

We first assessed the relation between differential 5mC clusters and known chromatin structural domains. We used high-resolution chromosome conformation capture (Hi–C) maps of neural progenitor cells since topologically associating domains (TADs) are relatively conserved between cell types, despite some genome reorganization occurring during differentiation^45^. All the main differential 5mC clusters were located within TADs identified by Bonev et al.^45^, but were smaller, and appeared to coincide with sub-TADs (Fig. 3f, Supplementary Fig. 3a–f). Importantly, these clusters contained D1/D2 differentially expressed genes, and the 5mC was higher in the cell type with lower expression of these genes. These observations indicate that the main differences in 5mC are found within clusters that map within structural domains and contain differentially expressed genes of potentially key importance for D1/D2 differences and striatal function.

### 5mC and 5hmC D1/D2 differences are mostly found in coding gene bodies

**Fig. 4:**
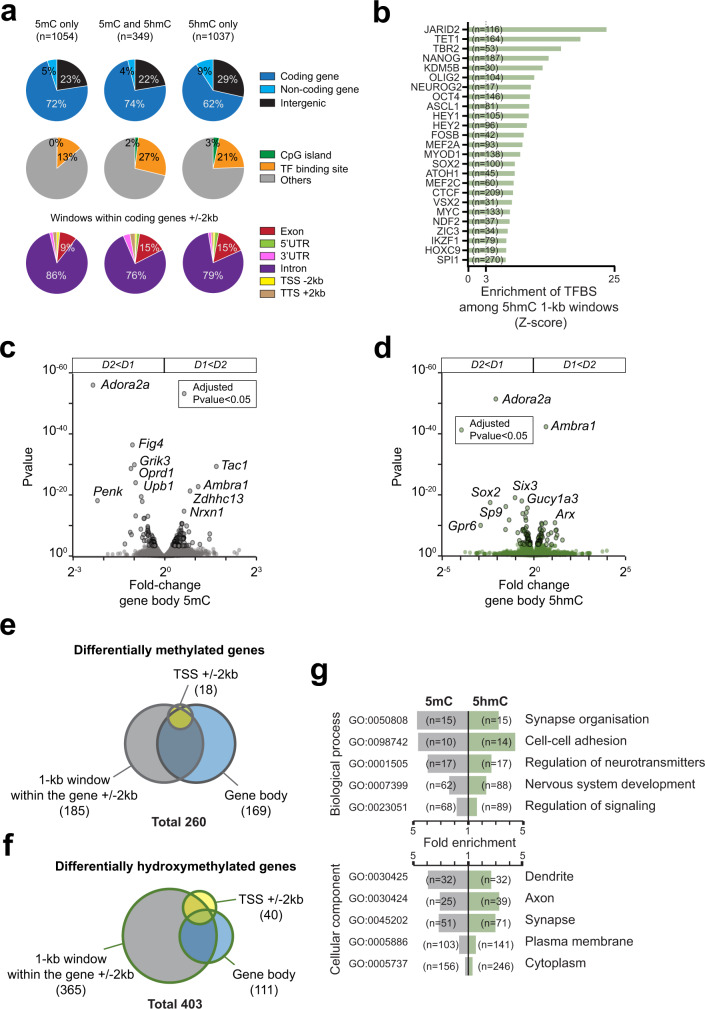
D1/D2 differences in 5mC and 5hmC are mostly found in genes. **a** Overlap of D1/D2 5mC and 5hmC significantly different 1-kb windows with genomic features. **b** Top 25 TFBSs enriched among the significant 1-kb 5hmC windows. **c** Volcano plot of gene body 5mC differences. **d** Volcano plot of gene body 5hmC differences. **e** Venn diagram of genes with 5mC differences found by the TSS, gene body, or 1-kb windows analyses. **f** Same as in (**e**) for 5hmC. **g** Enriched gene ontology terms (FDR < 3 × 10^−2^ for biological processes and FDR < 2 × 10^−2^ for cellular components) for differentially methylated (*n* = 258) or hydroxymethylated (*n* = 380) genes, identified with the 1-kb window, the TSS, or the gene body analyses.

We then examined the location of the DNA modification differences. The observation that most of the differentially methylated clusters include several genes that show differential expression led us to examine in more detail whether the 5mC and 5hmC differences might correspond to specific genes, their bodies, or their regulatory elements. We first assessed whether the 1-kb windows of D1/D2 differences overlapped specific genomic features (Fig. 4a). Remarkably, even though the genic regions represent only a small proportion of the genome, most of the 5mC and 5hmC differences (72% and 62%, respectively) were located within coding genes, overlapping 185 and 365 genes, respectively (Supplementary Tables 8, 9). The vast majority of the differences were found within gene bodies (introns and exons). We also used the *OReGanno*^46^ and *Cistrome*^47^ databases to assess whether differentially modified regions encompassed TFBSs. We observed that differential 5hmC windows overlapped TFBSs more often than the differential 5mC windows (Fig. 4a, b, Supplementary Fig. 4a). Interestingly, differential 5hmC windows were also enriched in TET1 sites (Fig. 4b), an enzyme that catalyzes the oxidation of 5mC into 5hmC. It should be noted that we probably underestimate the percentage of targeted TFBS since important ones are missing in databases (e.g., SP9 important for D2-SPNs differentiation^30^).

As most of the differences for both DNA modifications were localized within gene bodies, we identified the differentially modified genes (from transcription start site [TSS] to transcription termination site [TTS], see Methods). We found 169 genes that differ for global gene body 5mC content between D1 and D2, (Fig. 4c, Supplementary Table 10), including genes characterized for their functions in D1-SPNs (e.g., *Tac1*^48^) or D2-SPNs (e.g., *Adora2a*^49^ and *Penk*^50^). For 5hmC, we found 111 genes with differential gene body modification (Fig. 4d, Supplementary Table 11), including 53 of the differentially methylated genes (e.g., *Adora2a, Ambra1*) that were generally more methylated and hydroxymethylated in the same cell type (Supplementary Fig. 4b). Differentially hydroxymethylated genes included some genes encoding TFs necessary for striatal development, such as *Sp9*^51^ and *Six3*^31^, and other TFs of unexplored function to date in SPNs (e.g., *Sox2*, *Arx*). We also analyzed 5mC and 5hmC differences at the TSSs (±2 kb) of genes and found 18 significantly different genes for 5mC (Supplementary Fig. 4c, Supplementary Table 12) and 40 for 5hmC (Supplementary Fig. 4d, Supplementary Table 13).

Overall, a total of 260 genes displayed a significant D1/D2 difference in 5mC, either at the TSS, at the gene body level, and/or more locally in a 1 kb window within the gene body ±2 kb (Supplementary Table 14). Comparison of these various analyses showed that most of the 5mC differences between D1 and D2 were detected by total gene body comparison or 1-kb windows analysis (Fig. 4e). Hydroxymethylation was significantly different in 403 genes either at the TSS, at the gene body level, and/or more locally in a 1 kb-window within the gene body ±2 kb (Supplementary Table 14). These 5hmC differences were more local than 5mC differences, mostly detected by the 1-kb windows analysis (Fig. 4f), in accordance with their enrichment at TFBSs as reported in other cell types^52,53^. The differentially modified genes were predominantly related to neuronal-specific functions (Fig. 4g), similar to what we observed for gene expression. Thus, analyses of the D1/D2 differences show that differentially methylated and hydroxymethylated regions are highly enriched in genic regions. Hydroxymethylation frequently overlaps with TFBS, indicating a possible link with differential gene expression.

### Differential 5mC and gene expression are inversely related

**Fig. 5:**
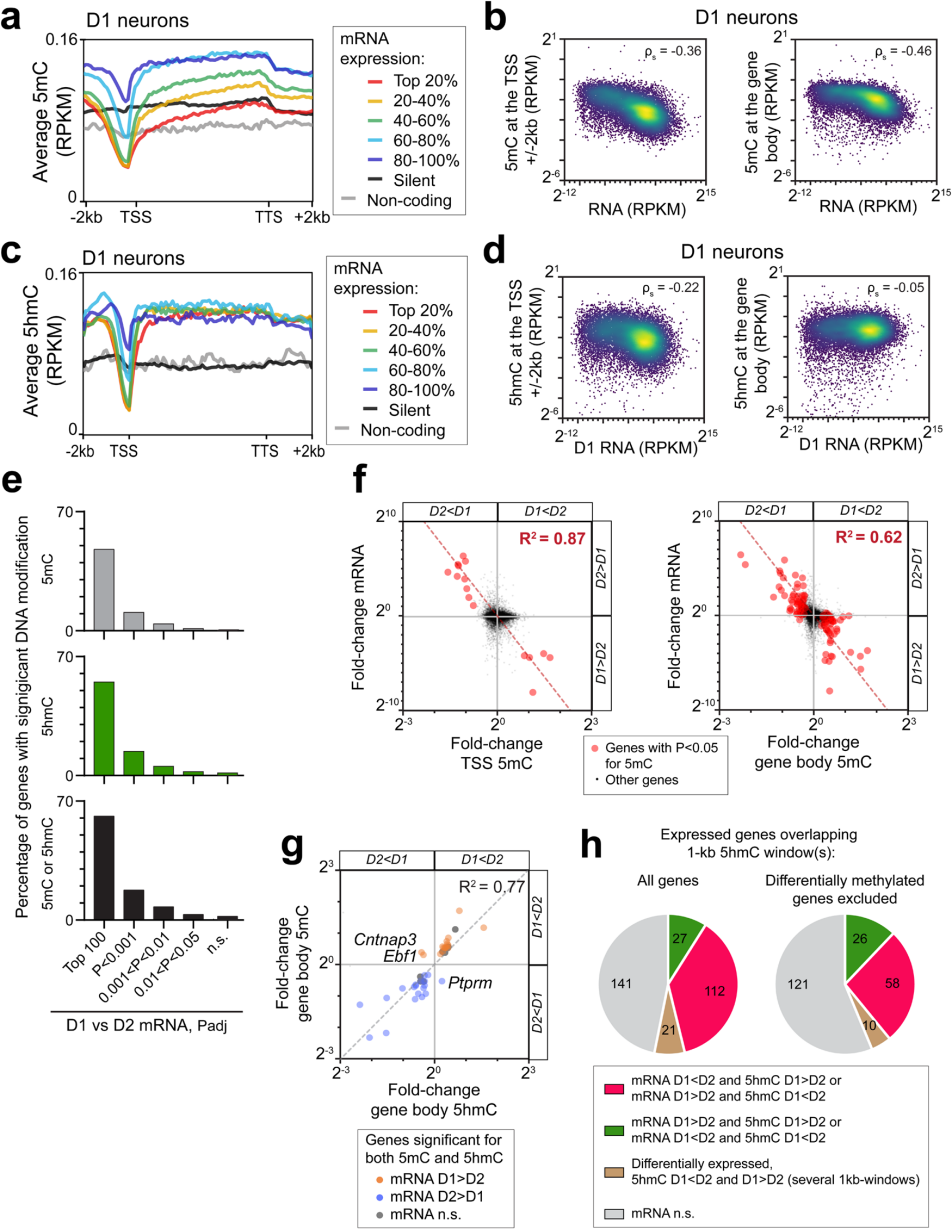
Relation between gene 5mC and 5hmC, and mRNA levels. **a** Metagene profiles of 5mC according to gene expression levels in D1 neurons. In these plots the gene body lengths are normalized while the preceding and following 2-kb are linear. **b** Density plots of 5mC levels at TSS or gene body, as a function of mRNA levels for expressed coding genes in D1 neurons. ρ_s,_, Spearman correlation coefficient. **c**, **d** Same as in (**a**, **b**) but for 5hmC. **e** Percentages of D1/D2 differentially expressed genes showing 5mC, 5hmC, or both differences (found by the TSS, gene body, or 1-kb windows analyses), depending on the significance of mRNA differences. **f** Scatter plots of the D1/D2 differences in mRNA levels as a function of the changes in 5mC at TSS or gene body. The linear regressions are shown. **g** Scatter plot of 5hmC changes compared to 5mC changes for genes with significant D1/D2 differences for both. **h** Expression changes of genes overlapping at least one 1-kb window significantly different for 5hmC between D1 and D2 neurons.

We first looked for a global relationship between gene expression and DNA modifications in D1- and D2-SPNs. We compared the 5mC at the TSS and gene bodies with the translatome ranked as quintiles of gene expression (Supplementary Fig. 5a) and found a global trend of higher gene expression associated with lower 5mC in both D1- (Fig. 5a) and D2-SPNs (Supplementary Fig. 5b). This pattern was similar to that in other neurons but contrasted with non-neuronal cell types^2,9^. We observed a relative decrease in 5mC upstream of the TSS for all expressed genes, whereas the 5mC profile of silent protein-coding or non-coding genes was flat, and low for the latter, in agreement with previous studies^2,9^. Despite these general trends, 5mC levels at the TSS and gene bodies were variable between genes and not predictive of expression levels on a gene per gene basis (Fig. 5b, Supplementary Fig. 5c). When we did the same analyses for 5hmC, the most expressed genes displayed a drop in 5hmC at the level of the TSS, which was less pronounced in less expressed genes (Fig. 5c, Supplementary Fig. 5b). In contrast, no systematic differences among expressed genes was observed in gene body regions, which showed consistently high levels of 5hmC, as observed previously in some neuronal types^9^. This illustrates the differences between 5mC and 5hmC distribution patterns, and indicates that 5hmC is less associated with decreased gene expression than 5mC. No change in 5hmC was observed along the TSS or gene body regions for silent coding or non-coding genes. The 5hmC levels were also very variable between individual expressed genes and were not predictive of mRNA expression levels (Fig. 5d, Supplementary Fig. 5c).

We then assessed the relationship between the differences in DNA modifications and gene expression between D1 and D2 samples. Overall, 11% of the differentially expressed genes carried significant differences in DNA modifications (Supplementary Fig. 5d). Among the top 100 genes more expressed in either D1- or D2-SPNs (excluding the genes in the BACs), 60 carried significant differences in either DNA modifications, with 46 differentially methylated and 53 differentially hydroxymethylated (Fig. 5e). Hence, both DNA modifications appear to be highly relevant to SPNs identities. Among the genes differentially methylated between D1 and D2 neurons, there was a strong negative correlation between the 5mC fold-changes at the TSS or gene body, and mRNA changes (Fig. 5f). This indicated that in the D1/D2 comparison, a decrease in DNA 5mC at the TSS and/or gene body is a good predictor of increased expression. The results suggest an involvement of 5mC at both the TSS and gene body in regulating differential gene expression between D1/D2 neurons.

### Local 5hmC is associated with differential expression in either direction

We then focused on D1/D2 5hmC differences and investigated their relation with translating mRNA levels. In genes differently hydroxymethylated in D1 and D2 neurons, we observed an inverse correlation of TSS or gene body 5hmC with mRNA levels (Supplementary Fig. 5e). However, these correlations were less tight than for 5mC. D1/D2 differences in DNA 5mC and 5hmC in gene bodies were generally correlated (Fig. 5g), with three exceptions (*Cntnap3*, *Ebf1*, and *Ptprm*) in which 5hmC levels were higher in the cell type in which 5mC was lower and expression higher. Because most of the 5hmC differences were local and found through the 1-kb window analysis (see above, Fig. 4f), we assessed whether the genes showing these local differences were also differentially expressed. Among the genes including at least one 1 kb-window significant for 5hmC D1/D2 difference, 53% were also differentially expressed between D1 and D2 neurons (Fig. 5h). However, these differences went in either direction, with 37% less expressed in the cell population in which they were more hydroxymethylated, 9% more expressed, and the remaining 7% containing 5hmC changes in both directions. Because we had found that 5hmC correlates with 5mC, and to exclude the possibility that associated 5mC could explain the association of 5hmC with gene repression observed here, we excluded the genes also differentially methylated at the gene body and/or TSS and found similar results. Thus, local 5hmC differences can be associated with either up- or down-regulation of gene expression, in line with its possible role in regulating TFBS suggested above.

### Whole-genome asymmetry of DNA 5mC and 5hmC

**Fig. 6:**
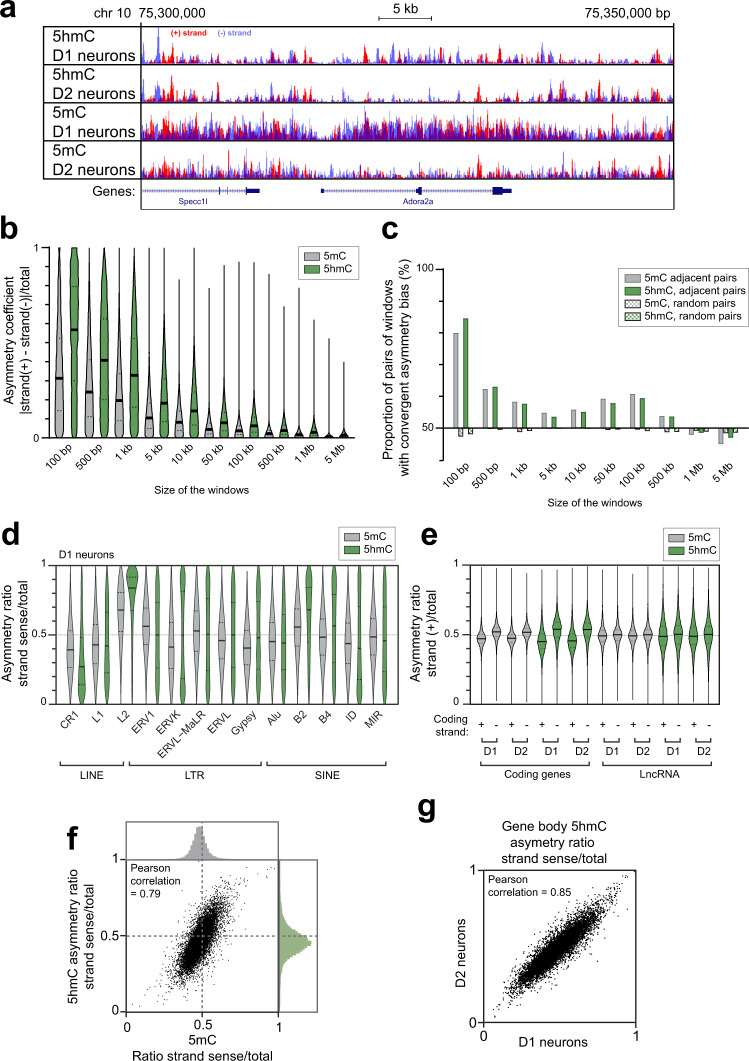
Strand asymmetry of DNA modifications. **a** Example of genome browser view of 5hmC and 5mC strand distribution in D1 and D2 neurons. Peak sizes correspond to the number of reads on the (+) strand and (−) strand. **b** Asymmetry coefficient of 5mC and 5hmC depending on the size of the windows considered. Random sampling of 10,000 windows for each size (see Methods). **c** Evaluation of the size of the asymmetric regions of 5mC and 5hmC. For each window size, a random sampling of 10,000 windows was chosen, and compared to the adjacent window or to a random window on the genome. Pairs are considered convergent when they both have more reads on the same strand [(+) or (−)]. **d** Genome-wide distribution of strand asymmetry of 5mC and 5hmC in repetitive transposable elements in D1 neurons. **e** Genome-wide distribution of strand asymmetry of 5mC and 5hmC in coding genes and lncRNA genes. **f** Correlation of 5hmC and 5mC asymmetry ratios in gene bodies. **g** Correlation of 5hmC asymmetry ratios in gene bodies between D1 and D2 neurons. In **b**, **d**, **e**, horizontal solid lines are medians and dotted line quartiles.

Our MeDIP and hMeDIP data allowed us to distinguish the 5mC/5hmC levels on each DNA strand. When we examined the two strands separately, we observed an asymmetry of DNA modifications that was more pronounced for 5hmC than for 5mC throughout the genome in both D1- and D2-SPNs (e.g., Fig. 6a, Supplementary Fig. 6a). The asymmetry was higher at smaller scales, although it was still present in intervals larger than 100 kb (Fig. 6b). In order to estimate the size of the asymmetric regions, we tested whether pairs of adjacent windows of a given size would have an asymmetry bias for the same strand more frequently than by chance. If so, that indicates that the tested windows are smaller than the typical size of the asymmetric regions. We observed that both 5mC and 5hmC displayed the same pattern, with a bimodal distribution indicating the existence of asymmetrically modified regions with nested sizes typically smaller than 5 kb and bigger ones up to 1 Mb (Fig. 6c).

We assessed the asymmetry distribution with respect to identified regions, including genes and retrotransposons, which represent >40% of the genome. All retrotransposons were particularly asymmetric, and different families of transposons displayed different strand asymmetry (Fig. 6d, Supplementary Fig. 6b). Repetitive transposable elements were particularly asymmetric, including LINE-1 elements, which are known to be active in neurons^54^, but also inactive elements (e.g., LINE-2).

We also observed that coding genes had more 5hmC on the template (i.e., non-coding) strand (Fig. 6e), but not in neighboring intergenic regions (Supplementary Fig. 6c). In contrast, long non-coding RNAs (lncRNAs) had globally similar levels of DNA modifications on both strands (Fig. 6e). Although strand asymmetry was less pronounced for 5mC than for 5hmC, the asymmetries of 5mC and 5hmC were highly correlated (Fig. 6f). The asymmetry on the genes was more consistent between replicates for 5hmC than for 5mC (Supplementary Fig. 6d) and was highly correlated between D1 and D2 neurons (Fig. 6g), with no significant difference between the two populations. DNA modification asymmetry did not depend on GC content or gene size (Supplementary Fig. 6e, f), but showed some degree of correlation with the strand ratio of CH (Supplementary Fig. 7a). Our data thus revealed a profound strand asymmetry of DNA modifications, in retrotransposons and coding genes, predominantly for 5hmC, and with a consistent organization between the two types of SPNs.

### Asymmetric gene body 5hmC is associated with higher gene expression

**Fig. 7:**
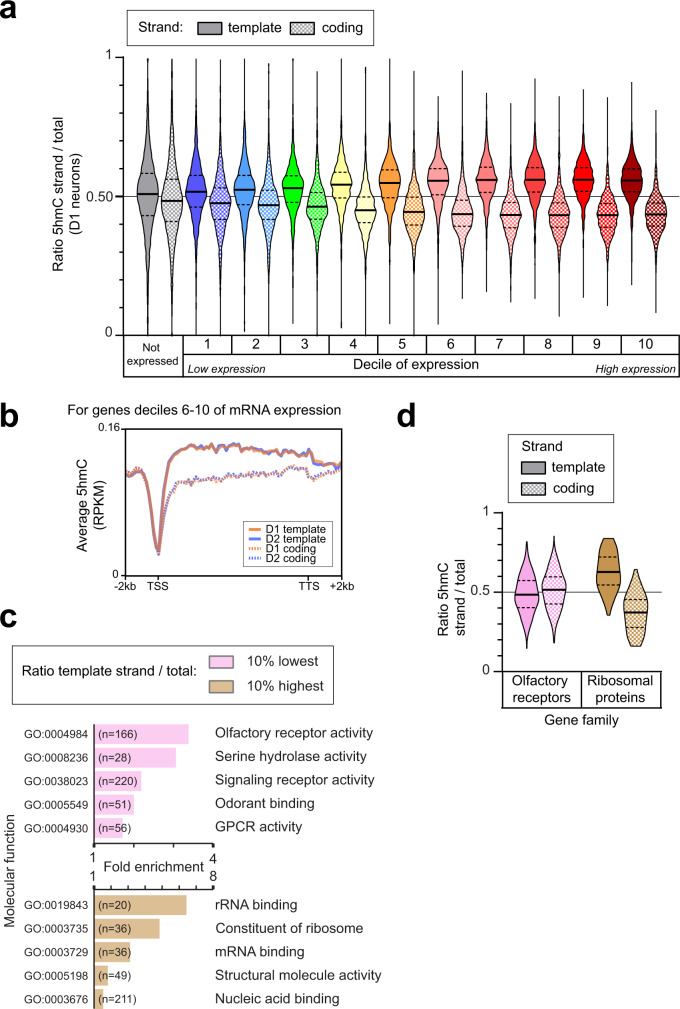
5hmC asymmetry is associated with gene expression. **a** Violin plots of the 5hmC asymmetry ratios in D1 neurons on the template and coding strands of coding genes with different expression levels. **b** Metagene profiles of 5hmC on the template and coding strands, for the highly expressed genes (top 50% of expressed coding genes). **c** Enriched gene ontology terms (molecular function, FDR < 2 × 10^−2^) for genes with the 10% lowest or 10% highest 5hmC ratio of the template strand. **e** Violin plots of the 5hmC asymmetry ratios on template and coding strands, for olfactory receptors genes and ribosomal proteins. In **a** and **d**, horizontal solid lines are medians, and dotted lines are quartiles.

Since modification asymmetry was high in coding genes, we asked whether the degree of 5hmC asymmetry was related to gene expression levels by comparing the strand asymmetry ratios for genes ranked by expression levels deciles (Fig. 7a, Supplementary Fig. 7b). Non-expressed genes displayed almost no asymmetry (median ratio 0.51 for the template/total), whereas the median ratio increased with expression and reached a plateau for the most expressed deciles. We checked the asymmetry of strand composition in relation to transcription levels and observed a similar bias in CH for the template strand, although less pronounced than 5hmC (Supplementary Fig. 7c). 5hmC asymmetry increases on average with the degree of gene expression, it is highly variable from gene to gene and therefore not predictive of expression on a gene per gene basis. The excess of 5hmC on the template strand was detected on gene bodies of highly expressed genes, but not at the TSS, where 5hmC on both strands dropped to the same low level (Fig. 7b). We did not detect differences in strand asymmetry for D1/D2 differentially expressed genes.

We then examined which genes were most represented among those with a high degree of 5hmC asymmetry (Fig. 7c) and identified highly expressed genes, such as those coding for ribosomal proteins. These genes were overrepresented among the 100 genes with the highest asymmetry ratio, and the whole family of ribosomal protein genes had a very high strand asymmetry (0.63 template/total, Fig. 7d). In contrast, olfactory receptor genes, which are virtually not expressed in the striatum, had particularly low ratios (0.48, Fig. 7c, d). Overall, our data reveal a link between DNA 5mC and 5hmC strand asymmetry and the degree of gene expression, which is much stronger in the case of 5hmC.

## Discussion

In this study, we compared the methylomes, hydroxymethylomes, and translatomes of two types of very closely related neurons, the D1- and D2-SPNs, characterized by the expression of dopamine D1 and D2 receptors, respectively. Previous reports on methylomes compared non-neuronal cells and neurons, or distant neuronal types^2,9,34–38^. Here, the comparison between these two homogeneous and transcriptionally similar populations sheds light on the regulation and influence of cytosine modifications in the last steps of differentiation towards specialized neurons. We found that the two cytosine modifications display similarities and differences in their localization, relation to transcription, and distribution between the two DNA strands.

Methylation differences between D1- and D2-SPNs are grouped in clusters with lower 5mC in one or the other population, including fifteen large regions in the genome (>100 kb). These clusters include genes differentially expressed between D1 and D2 neurons, and more expressed in the population in which they are less methylated. These differential 5mC clusters contain differentially expressed genes characteristic of D1- or D2-SPNs identities or important for their respective functions (e.g., *Adora2a*^49^, *Chrm4*^55^, *Penk*^50^, *Slc35d3*^56^*, Gpr6*^57^, *Gpr52*^58^, and *Nrxn1*^59^). They also contain other genes that have been linked with striatal-related pathologies (e.g., *Lingo2*^60^, *Nrxn2*^61^, *Mdk*^62^, *Grik3*^63^, *Fig4*^64^), as well as genes not previously identified as characterizing the two SPN types (e.g., *Dgkz*, *Ptprm*, and *Sntg2*). We suggest that these latter genes may also have important cell type-specific roles in striatal development and/or physiology. The extent of large 5mC clusters coincided with structural sub-domains within previously identified TADs. It is possible that 5mC is removed (or deposited) locally at some anchor points with the contribution of specific TFs^65^ and that the modification spreads within a chromatin sub-domain. Alternatively, the transcriptional activity within the sub-domain during neuronal development could prevent DNA methyltransferase DNMT3A activity^66^ which has been reported to play a role in depositing DNA 5mC de novo^67^ and is essential for neuronal maturation^68^. The existence of 5mC differences spanning several genes and intergenic regions (e.g., Fig. 3f) supports the first hypothesis, although, the second possibility is more in line with the preferential localization of 5mC differences to gene bodies (Fig. 4a). It is possible that the two scenarios are combined in various proportions.

DNA 5mC at gene TSSs is largely recognized as a repressive mark, whereas its role in gene bodies is more elusive. Gene body 5mC is positively correlated with transcription during development and has been proposed to facilitate transcription elongation^8^. In contrast, studies of various types of neurons and Bergmann glia have reported lower 5mC across the most highly expressed genes^2,9,34,36^. In SPNs, we similarly observed an inverse correlation between gene body 5mC and mRNA expression, although the levels of 5mC were very variable on a gene-per-gene basis, as in other cell types. In contrast, in the D1/D2 comparison at the single gene level, a decrease in gene body 5mC in one population was actually a predictor of increased expression in this population. Hence, our data confirm and refine the association between both TSS and gene body 5mC with gene repression in SPNs and, presumably, in other fully differentiated neurons. The fact that gene body 5mC correlates with repression in neurons, unlike other cell types, has been suggested to originate from the presence of CH 5mC on gene bodies in neurons, but it can also be linked to the specific high abundance of the methylated DNA-binding protein MECP2 in neurons^9,69^, which participates in transcriptional repression^70^.

We found that DNA 5hmC differences between D1 and D2 neurons were partly correlated to 5mC differences, but they were more scattered, in clusters of smaller size, and frequently overlapped TFBSs. Even though 5hmC is an intermediate for demethylation^4,5^, we observed that the 5mC and 5hmC profiles are distinct, and not related to transcription in the same manner, in agreement with previous observations. An inverse correlation was consistently found between 5mC and transcription, while this does not hold true for 5hmC. For example, an overall positive correlation between gene body 5hmC and gene expression was found for some brain cell types^9^. Our data show that differential 5hmC mostly affects TFBSs and that these localized 5hmC differences are associated with gene expression differences. In line with this, 5hmC has been shown to influence TF binding^71^. Thus, 5hmC has the potential to fine-tune the expression of particular genes, by influencing the binding of TFs.

Interestingly, we observed an asymmetry of methylation and 5hmC between the two strands of DNA genome-wide. Some degree of asymmetry was previously observed using bisulfite-based methods^72–74^, but enrichment-based single-strand methods MeDIP and hMeDIP allowed us to further characterize and probe the extent of the asymmetries. Our data reveal a widespread asymmetry over the genome, of small-size regions (5 kb or less) nested in bigger regions up to 1 Mb. The asymmetry of 5hmC is more pronounced than that of 5mC and is strongly associated with expression levels. Indeed, highly expressed genes such as ribosomal protein genes typically have more 5hmC on the template strand, whereas silent or lowly expressed genes have rather balanced 5hmC.

The asymmetry of 5hmC could be simply explained if 5hmC would occur in the CH context, where only one strand can be hydroxymethylated, rather than CG, where cytosines in both strands can be hydroxymethylated^75^. However, previous bisulfite-based studies have shown that 5hmC is found mostly in a CG context, although not exclusively^2,76^, and this would suggest that the 5hmC asymmetry is not directly dependent on base composition. Still, at the level of coding genes, we observed a correlation between CH and 5hmC asymmetries. Whether or not the CH content would be sufficient to explain the 5hmC asymmetry remains an open question, and would have to be investigated at the single-base level using bisulfite-based methods with extensive coverage.

Hydroxymethylation asymmetry in neurons could have functional consequences, notably for chromatin structure. Studies on synthetic (symmetric) hydroxymethylated DNA have shown that 5hmC can either enhance or decrease strand separation and affect the conformation of the double helix^77^. The 5hmC hydroxyl group can participate in hydrogen bonding, causing higher solvation energy^78^ and stabilizing DNA–protein interaction^79^. Therefore, the distribution of 5hmC on DNA has the potential to participate in chromatin conformation and/or stabilization in neurons where 5hmC is particularly abundant. Transposable elements are known to be enriched in DNA modifications^80^, including 5hmC^81^, and contain strand-specific methylation in non-CpG context^75^. We found marked strand asymmetry in retrotransposons, for 5mC and even more for 5hmC. Transposons play a role in chromatin organization^82^, and the consequences of asymmetrical 5hmC on DNA conformation remain to be investigated. In transcribed genes, 5hmC could favor strand separation and facilitate transcription. Our data may suggest that asymmetry in transcribed genes is not a direct consequence of transcription, since differentially expressed genes between D1 and D2 do not show asymmetry differences.

Our work uncovers specific features of DNA modifications in neurons and characterizes the DNA 5mC and 5hmC differences between the two major populations of SPNs. It highlights the importance of DNA modifications in distinguishing the most differentially expressed genes in the two major populations of dopamine target cells in the striatum. Some of the key TFs for SPNs differentiation are differentially methylated or hydroxymethylated, such as *Ebf1* and *Sp9*. Differentially modified regions include yet uncharacterized genes that have the potential to be highly relevant for the identities or function of D1 and D2 neurons. The characterization of epigenetic differences between D1 and D2 SPNs also provides the necessary background for assessing their possible alterations in physiological and pathological conditions that differentially affect these two populations, including reinforcement learning, drug addiction, Huntington’s disease, and Parkinson’s disease. Genes identified in this study might be used as particular targets and contribute to the development of new therapeutic strategies in striatum-related pathologies.

## Methods

### Animals

BAC transgenic mice that express enhanced green fluorescent protein fused to the N-terminus of the large subunit ribosomal protein L10a under the control of dopamine D1a or D2 receptor promoter (*Drd1-EGFP-L10a* or *Drd2-EGFP-L10a*), generated as described^26^, were maintained as heterozygotes on a C57Bl/6J background. Experiments with both lines were run in parallel. All the experiments were in accordance with the National Institutes of Health Guide for the Care and Use of Laboratory Animals and approved by Rockefeller University’s Institutional Animal Care and Use Committee. For all experiments, male and female mice were 2-3 month-old, generated by in vitro fertilization (Transgenic and Reproductive Technology Center, Rockefeller University). Animals were housed on a 12-h light-dark cycle, in stable conditions of temperature, with food and water *ad libitum*. Four mice were pooled for each sample, males and females were mixed, and the sex ratio was counterbalanced between groups. All conditions were run in triplicates.

### Tissue preparation

Each sample consisted of whole striata from 4 mice. Each mouse was slightly anesthetized with CO_2_ before being decapitated. Striata from both hemispheres were dissected and placed into ice-cold Hank’s Balanced Salt Solution 1× (Invitrogen 10× solution: 12.6 mM CaCl_2_, 4.92 mM MgCl_2_, 4.07 mM MgSO_4_, 53.3 mM KCl, 4.41 mM KH_2_PO_4_, 1380 mM NaCl, 3.36 mM Na_2_HPO_4_, 55.6 mM D-glucose) containing 2.5 mM HEPES-KOH pH 7.4, 35 mM glucose, 4 mM NaHCO_3_ and 100 µg.mL^−1^ cycloheximide. When the striata from the 4 mice of the same sample had been collected, they were placed in a 2-mL Dounce homogenizer (Dominique Dutscher, Brumath, France) containing 1 mL of homogenization buffer (20 mM HEPES-KOH pH 7.4, 5 mM MgCl_2_, 150 mM KCl, 0.5 mM DL-dithiothreitol, 100 µg.mL^−1^ cycloheximide, EDTA-free protease inhibitors (Roche), 400 U.mL^−1^ Superasin (Life Technologies), 200 U.mL^−1^ RNasin (Promega). Ten strokes of pestle A (clearance 76–127 µm) followed by 10 strokes of pestle B (clearance 12–63 µm) were applied gently to avoid damage to nuclei. The homogenate was then centrifuged at 2000 × *g* 10 min 4 °C. The pellet and supernatant were separated to proceed with nuclei sorting and mRNA recovery, respectively.

### Cell-type-specific mRNA recovery and sequencing

The supernatant was complemented with NP-40 (final concentration 1% vol/vol) and 1,2-diheptanoyl-sn-glycero-3-phosphocholine (final concentration 30 mM) and incubated for 5 min. It was then centrifuged at 20,000 × *g* for 10 min 4 °C and the pellet was discarded. Immunoprecipitation was performed according to previous protocols^26,83^. First, magnetic beads coated with anti-GFP antibody were prepared as follows: 300 µL of Streptavidin MyOne T1 Dynabeads (Invitrogen) per sample were washed in phosphate-buffered saline (PBS), incubated 35 min at room temperature (RT) with 120 µg of biotinylated protein L in PBS, washed 5 times with bovine serum albumin (BSA) 30 g.L^−1^ in PBS, incubated 1 h at RT with 100 µg of monoclonal anti-GFP antibodies (50 µg clone 19F7 + 50 µg clone 19C8, Memorial Sloan-Kettering Monoclonal Antibody Facility, New York) in the homogenization buffer containing 1% (vol/vol) NP-40, washed 3 times and finally resuspended in 200 µL of homogenization buffer complemented with 1% (vol/vol) NP-40.

Magnetic beads coated with anti-GFP antibodies were added to the homogenates. After the addition of Superasin (final concentration 200 U.mL^−1^, Life Technologies) and Rnasin (final concentration 400 U.mL^−1^, Promega), the samples were incubated for 16 h at 4 °C under gentle end-over-end rotation. After 4 washes with homogenization buffer complemented with 1% (vol/vol) NP-40 and 200 mM KCl (total concentration KCl 350 mM), the RNA was eluted with RLT Plus buffer from the RNeasy Plus Micro kit (Qiagen) and 10 µL.mL^−1^ β-mercaptoethanol (10-min incubation at RT and vortex). Then the RNA was purified according to the manufacturer’s instructions, with an on-column DNAse-I digestion step. The quantity of RNA was determined by fluorimetry using the Quant-iT Ribogreen, and its integrity was checked using the Bio-Analyzer Pico RNA kit before library preparation.

Ten nanograms of RNA were used for reverse transcription, performed with the Nugen Ovation RNAseq v2 kit. cDNAs were quantified by fluorometry, using the Quant-iT Picogreen reagent, and ultra-sonicated using a Covaris S2 sonicator with the following parameters: duty cycle 10%, intensity 5, 100 cycles/burst, 5 minutes. Two hundred nanograms of sonicated cDNA were then used for library construction using the Illumina TruSeq RNA sample prep kit, starting at the End-Repair step, and following the manufacturer’s instructions. The libraries were quantified with the Bio-Analyzer High-sensitivity DNA kit, multiplexed, and sequenced on an Illumina HiSeq 2500 instrument. We obtained more than 40 million 50 bp paired-end reads per sample.

### Cell-type specific DNA recovery

The pellet containing the nuclei (obtained after the 2000 × *g* centrifugation) was resuspended in a density solution with 29% iodixanol, prepared as follows: five volumes of Optiprep^TM^ (Sigma Aldrich) containing 60% iodixanol were mixed with one volume of 150 mM KCl, 30 mM MgCl_2_ and 120 mM Tris, pH 7.4. This 50% iodixanol solution was then further diluted to make a solution containing 29% iodixanol, using 250 mM sucrose, 25 mM KCl, 5 mM MgCl_2_, and 20 mM Tris, pH 7.4, as a diluent. The pellet was dissociated by gently pipetting up and down 15 times. The homogenate in the 29% iodixanol solution was centrifuged at 10,000 × *g* 30 min 4 °C (swinging buckets, TLS55 rotor, Beckman TL-100 ultracentrifuge). The nuclear pellet was resuspended in 250 mM sucrose, 25 mM KCl, 5 mM MgCl_2_, 20 mM Tricine-KOH pH 7.4, 1% (vol/vol) donkey serum, 10 µM DyeCycle Ruby (Invitrogen). The rest of the sorting procedure was performed as in our previous study^84^. The preparation was sorted with a FACSAria (BD) cell sorter equipped with 640 and 488 nm excitation lasers and an 85 µm nozzle. Nuclei were gated by two criteria: the signal from DyeCycle Ruby corresponding to single nuclei and a GFP signal above background fluorescence (as assessed by comparison with nuclei from a wild-type littermate mouse). At least 100,000 nuclei were collected for each sample, with a purity >97%. Sorted nuclei in PBS were snap-frozen in liquid nitrogen.

### DNA fragmentation and ligation

DNA extraction was performed after proteinase K and RNase A treatment, using a phenol/chloroform standard protocol. Glycoblue^TM^ (Life Technologies) was added at the precipitation step to avoid subsequent pellet loss. DNA was then resuspended in 130 µL Tris pH 8.0. DNA was fragmented on an S2 Focused-ultrasonicator (COVARIS). The size of the fragments was assessed on a Bioanalyzer (Agilent Technologies) using a High Sensitivity DNA Kit. The mean size of the fragments was 150 bp for the MeDIP experiment and 250 bp for the hMeDIP experiment. Non-methylated TruSeq DNA adapters (synthesized by Sigma), with different indexes (for sample multiplexing before sequencing), were ligated using a SPRIworks Fragment Library System I kit (Beckman) on an SPRI-TE instrument, according to the Illumina Truseq DNA sample prep kit protocol.

### Hydroxymethylated/methylated DNA immunoprecipitation

The immunoprecipitation (IP) protocol was performed as follows, it was adapted from Weber et al.^85^, with some modifications, including the ligation step with Y-adapters, performed prior to IP, to increase the yield and allow strand-specificity. It was miniaturized, and for this purpose, 200-µL tubes were used. IP conditions were optimized beforehand using DNA from a similar number of non-GFP nuclei, which underwent all the previous steps, assessing the best conditions using a DNA 5mC control package (Diagenode). The obtained fragmented DNA with adapters in 80 µL water was denatured at 95 °C 10 min and quickly cooled on ice. All the immunoprecipitation steps were then performed at 4 °C to keep the DNA in single-strand conformation. The IP buffer was 10 mM sodium phosphate buffer pH 7.0, 150 mM NaCl, 0.5% (vol/vol) Triton X-100. For each sample, 1 µg of 5-methylcytosine monoclonal mouse antibody clone 33D3 (Diagenode Mab-081) was incubated for 2 h under gentle end-over-end rotation, then 10 µL of anti-mouse IgG-coated magnetic beads (which had been previously washed with BSA 10 g.L^−1^ in PBS and IP buffer) were added for overnight incubation (total volume 110 µL). After 3 washes with the IP buffer, the DNA fraction bound to the beads was eluted by a 3-h incubation at 37 °C in a solution containing 10 mM EDTA, 50 mM Tris pH 8.0, 0.5% (vol/vol) sodium dodecyl sulfate, and 250 µg.mL^−1^ proteinase K, with shaking. The immunoprecipitated DNA was then purified using a standard phenol/chloroform extraction.

For hMeDIP, the IP was performed as for the MeDIP, also in a single-strand conformation, except that only 5 µL of magnetic beads were used per sample, with 0.1 µg 5-hydroxymethylcytosine monoclonal mouse antibody (Diagenode Mab-31HMC).

Both 5mC and 5hmC antibodies were previously validated. Datasheets showing the specificity of the antibodies for either 5mC or 5hmC are available on the manufacturer’s website. These antibodies are not expected to show a preference for the CG or the CH context, as they were both generated using only a modified C as hapten. The IP conditions were optimized using the “5-hmC, 5-mC, and cytosine DNA standard pack for hMeDIP” (Diagenode, AF-107-0040). This kit contains hydroxymethylated, methylated, and unmethylated DNA standards, to ensure optimum conditions for the recovery of either methylated or hydroxymethylated DNA.

### Library preparation of immunoprecipitated DNA and sequencing

The immunoprecipitated DNA was amplified for 12 cycles and purified with AMPureXP magnetic beads (Beckman Coulter Genomics) to remove fragments smaller than 100 bp. After quantification using Qubit and quality assessment with a Bioanalyzer, libraries were mixed in equimolar proportions and sequenced on an Illumina Hiseq 1000 instrument, running a single read 50 bp protocol using the P5 primer. We obtained more than 100 million reads per sample for the MeDIP experiment and 50 million reads for the hMeDIP experiment.

### Strand specificity

The MeDIP and hMeDIP protocols included several specific adaptations: ligation of Y-unmethylated Illumina adapters right after fragmentation by sonication and before denaturation of the DNA and IP of single-stranded DNA. The choice to ligate the adapters prior to denaturation was originally done to improve the efficiency and use a limited number of sorted nuclei (100,000). In both MeDIP and hMeDIP experiments, DNA was denatured before immunoprecipitation of single strands, allowing us to distinguish the modification levels of each strand individually.

### Read alignment

The sequencing quality was checked with FASTQC software (0.10.1)^86^. The read library manipulations were performed using the FASTX-toolkit software suite (0.0.13). The reads were clipped according to their respective adapter sequence and trimmed according to their per base sequence quality for each library. The reads were aligned to the mm10 mouse downloaded from the UCSC genome using BWA (0.7.5)^87^ for MeDIP and hMeDIP libraries and Tophat (2.0.10) for the RNA libraries. The aligned libraries were filtered for mapping quality “-q 30” using Samtools (0.1.19). After these quality steps, there were, on average, 108 × 10^6^ reads per library for the MeDIP, 49.10^6^ reads per library for the hMeDIP, and 105 × 10^6^ reads for the RNA. Genomic views of read coverage were generated using Integrated Genomics Viewer tools and browsers (IGV 2.0; http://www.broadinstitute.org/igv/).

### Statistics and reproducibility

*P* values were computed using R packages, as detailed below for each specific analysis. Pearson and Spearman correlation coefficients were computed using the cor() function in R. Pearson correlation coefficients were computed to assess the reproducibility between replicates.

### Differential expression

Reads were assigned to protein-coding genes using Rsubread (v1.28.0). Differentially expressed genes were obtained using the Limma package with the voom function (3.36.5). A cutoff of adjusted *P*_value_ < 0.05 was used for differentially expressed genes. Detailed results from this analysis are supplied in Supplementary Table 1 and include *P* values and fold changes for the protein-coding genes.

### MeDIP and hMeDIP windows analyses

The differentially methylated and hydroxymethylated regions were assessed using the R Bioconductor package MEDIPS (v1.16.0)^88^, with several window sizes tested (100 bp, 500 bp, 1 kb, 2 kb, 8 kb, 20 kb, 50 kb). Results from the 1-kb analyses are supplied in Supplementary Tables 2 and 3. The complete lists of genes overlapping 1-kb windows with *p* < 0.01 are provided in Supplementary Tables 8 and 9.

### Clustering

To estimate the size of differentially modified DNA regions between D1- and D2-SPNs, we first grouped 1-kb windows, which were significantly differentially modified and were less than 5 kb from each other. We then included neighboring significant 1-kb windows as long as the density of significant windows in the cluster was above 1/8, to obtain the final clusters. We only grouped windows that were significant in the same direction (e.g., both more methylated in D2). Detailed results from these analyses are supplied in Supplementary Tables 4 and 6. The lists of the genes overlapping the largest clusters are provided in Supplementary Tables 5 and 7.

### Feature analysis

Differentially methylated or hydroxymethylated windows were assigned to the genomic features using Bedtools (2.29.2). The TFBSs used as a reference originated from the ORegAnno database^46^.

### Transcription factor analysis

Mouse TFBSs were downloaded from the Cistrome database^47^. For each TF, the overlap with significant 1-kb windows was quantified using Bedtools (2.29.2). In order to compute a z-score, the average random overlap and standard deviation were obtained by shuffling the binding sites intervals 100 times on the mouse genome.

### MeDIP and hMeDIP, TSS, or gene body analyses

Reads were assigned to genes or TSSs using Rsubread (v1.28.0). The differentially methylated or hydroxymethylated genes were obtained with the R Bioconductor package DESeq2 (v1.27.32)^89^ without independent filtering. A cutoff of adjusted *P*_value_ < 0.05 was used for differentially modified genes or TSSs. Detailed results from these analyses are supplied in Supplementary Tables 10–13 and include *P* values and fold changes for all the genes.

### Gene ontologies

Gene ontologies enrichment analysis was performed with the GO Consortium online tool (http://geneontology.org/)^90,91^, using all the *Mus musculus* genes in the database as the common reference list for all the analyses.

### Metagene

Metagene plots were done using the R package Metagene (v2.4.3).

### Venn diagrams

The Venn diagrams were built using the https://www.stefanjol.nl/venny resource.

### Strand-specific visualization

Bam files were split into Forward and Reverse reads, and replicates were merged using Samtools (0.1.19). Genomic views of read coverage were generated using IGV 2.0.

### Asymmetry coefficient of DNA modifications

To assess the distribution of the asymmetry coefficient at different window sizes, 10,000 random windows of each size were selected using Bedtools shuffle. The asymmetry coefficient was computed as follows: |number of reads on the (+) strand—number of reads on the (−) strand|/total number of reads. The windows with less than 20 reads total were excluded.

### Size of the asymmetric regions

To estimate the size of the asymmetric regions, we first selected 10,000 random windows of each size (from 100 bp to 5 Mb) using Bedtools shuffle. For each window, we determined which strand had more reads. We then assessed whether the downstream neighboring window of the same size had a bias towards the same strand or not. As a control, the windows were also compared to random windows on the genome. If adjacent pairs of windows of a given size tend to have a concordant bias compared to chance, it implies that the typical asymmetric regions are bigger than the size of the windows considered. The windows with less than 20 reads total were excluded.

### Asymmetry coefficient of the CH content

The R package Rtracklayer (v1.50.0) was used to import the bed file of the regions of interest. The full genome sequences were obtained from the package BSgenome.Mmusculus.UCSC.mm10 (3.15). We manipulated the genomic intervals with GenomicRanges (v3.15) and analyzed the DNA content with Bsgenome (v1.58.0).

### Reporting summary

Further information on research design is available in the Nature Portfolio Reporting Summary linked to this article.

## Supplementary information

Supplementary Information

Description of Additional Supplementary Files

Suppl Tables 1-14

Reporting Summary

## Data Availability

The sequencing data (FASTQ files) generated in this study have been deposited in the GEO database under accession code GSE186572. All other data that support this study are in the Supplementary Tables and available from the corresponding authors upon reasonable request.
