# Supplementary material for: DNA methylation and hydroxymethylation characterize the identity of D1 and D2 striatal projection neurons
Source: Commun Biol. 2022 Dec 1;5:1321. doi: 10.1038/s42003-022-04269-w (PMC9715678; doi:10.1038/s42003-022-04269-w)
Supplement: Supplementary file 1 — Supplementary Information [file 42003_2022_4269_MOESM1_ESM.pdf]

DNA methylation and hydroxymethylation characterize the identity of D1 and D2 striatal projection neurons

Supplementary figures

Lucile Marion-Poll, Jean-Pierre Roussarie, Lieng Taing, Cloelia Dard-Dascot, Nicolas Servant, Yan Jaszczyszyn, Emmanuelle Jordi, Eskeatnaf Mulugeta, Denis Hervé, Déborah Bourc'his, Paul Greengard, Claude Thermes, Jean-Antoine Girault

Supplementary Figure 1: D1- or D2-BAC-TRAP.

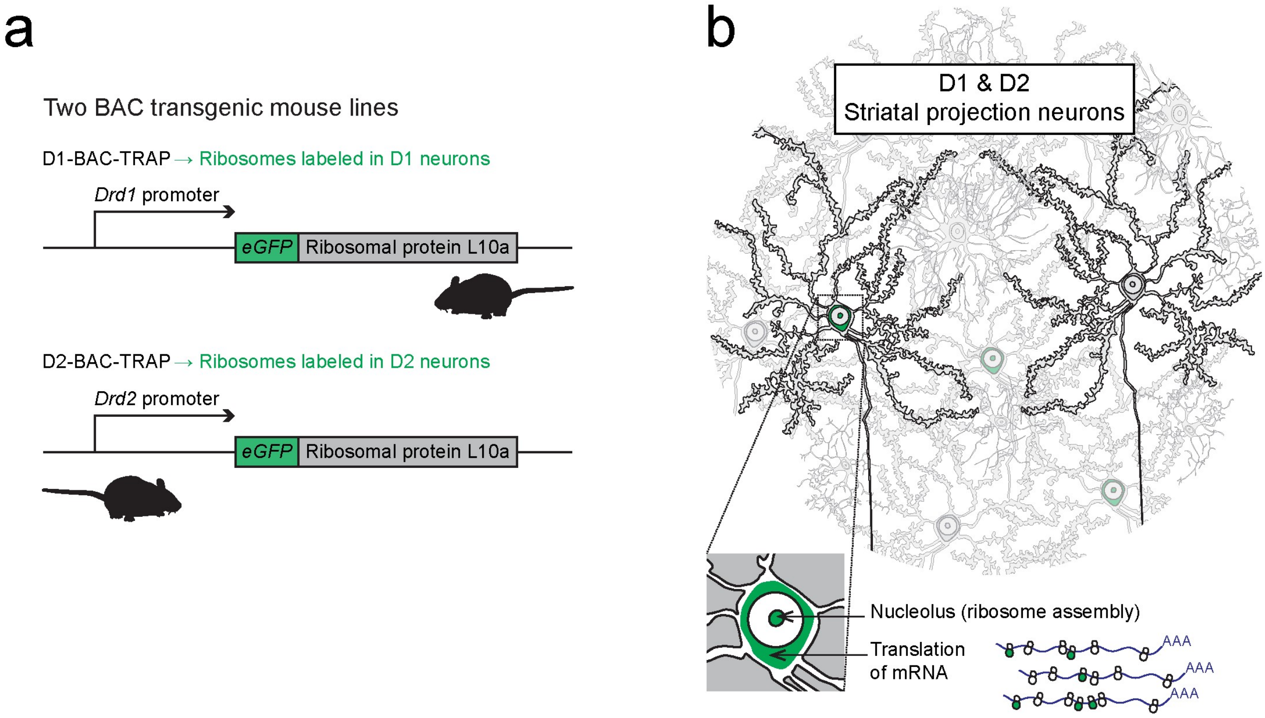

a Transgenic mouse lines used in this study. **b** Localization of EGFP-RPL10A in the nucleoli and on translating mRNA, in either D1 or D2 SPNs.

Supplementary Figure 2: FANS gating strategy and mRNA enrichment.

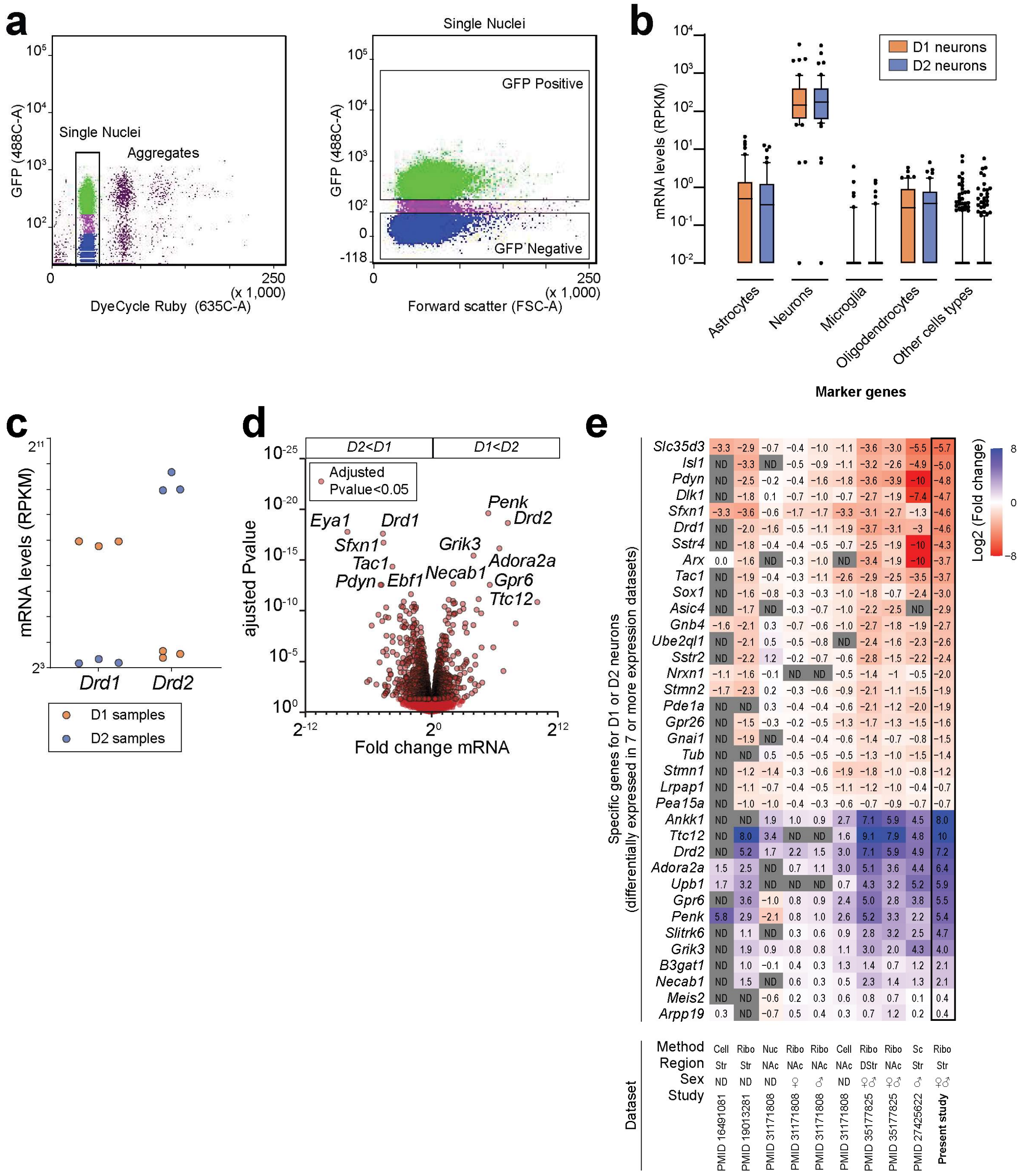

a Gating strategy for the sorting of GFP-positive single nuclei. DNA is labeled with DyeCycle Ruby. **b** mRNA levels of cell-type specific marker genes, previously identified by Gokce, et al. [16]. **c** *Drd1* and *Drd2* mRNA levels in D1 and D2 samples. **d** Volcano plot of mRNA differences. **e** Heatmap showing the RNA fold-change obtained by previous datasets[16,26,42-44] compared to the present study, for all the genes that were found significantly different between D1 and D2 SPNs in at least 7 datasets. Cell, whole cell sorting by FACS; Ribo, immunoprecipitation of ribosome-bound mRNAs; Nuc, nuclear sorting by FANS; Sc, single-cell; Str, striatum; DStr, dorsal part of the striatum; NAc, nucleus accumbens (ventral part of the striatum); ND, not determined.

Supplementary Figure 3: Position of differentially methylated and hydroxymethylated clusters within TADs.

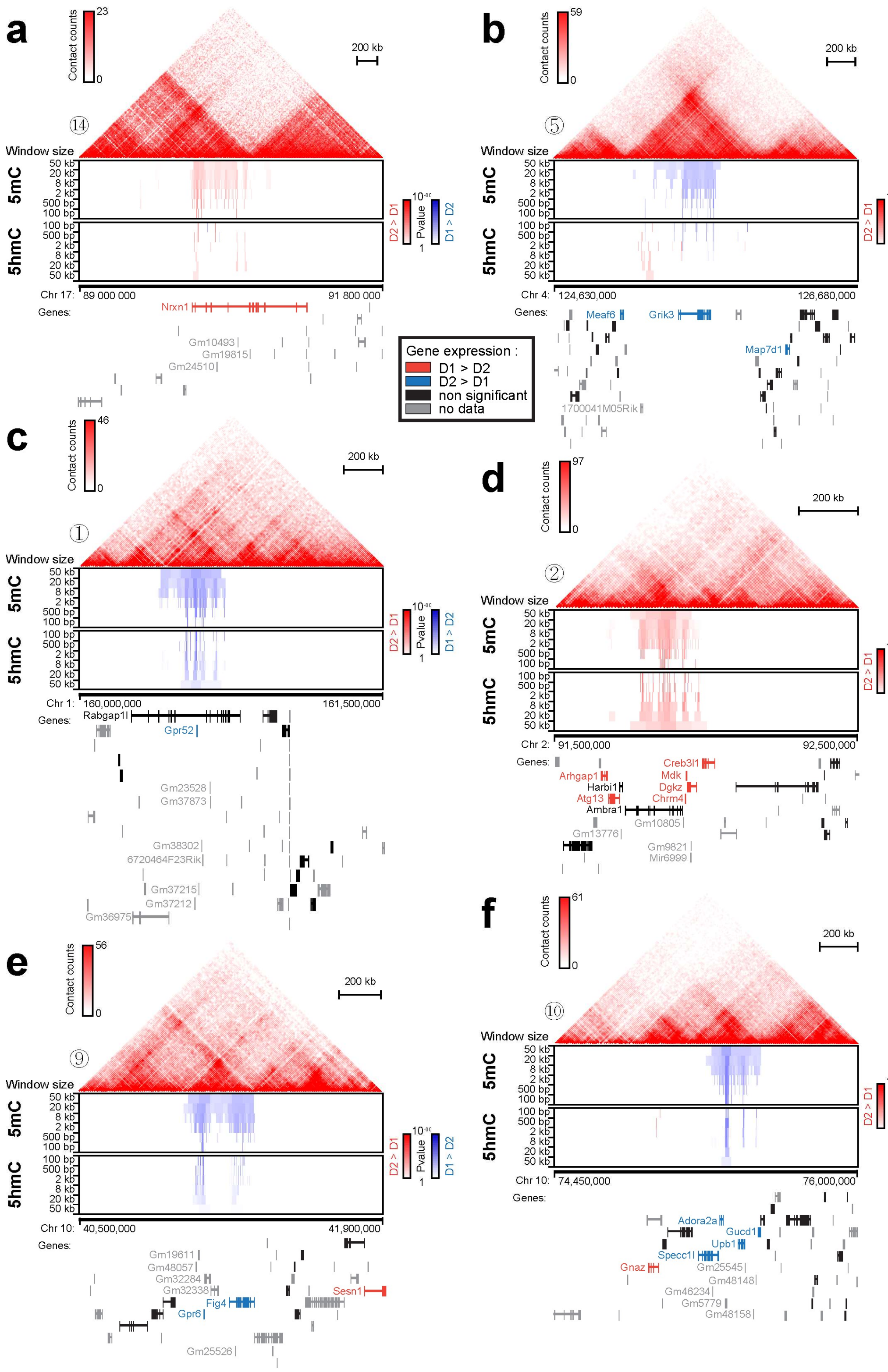

Hi-C interaction frequencies displayed as two-dimensional heat maps[45], superimposed with each cluster. The differentially methylated or hydroxymethylated regions were analyzed with multiple window sizes. The differentially expressed genes within each region are indicated. The cluster numbers are as indicated in Fig. 3a. **a** Cluster 14. **b** Cluster 5. **c** Cluster 1. **d** Cluster 2. **e** Cluster 9. **f** Cluster 10.

Supplementary Figure 4: D1/D2 differential 5mC and 5hmC.

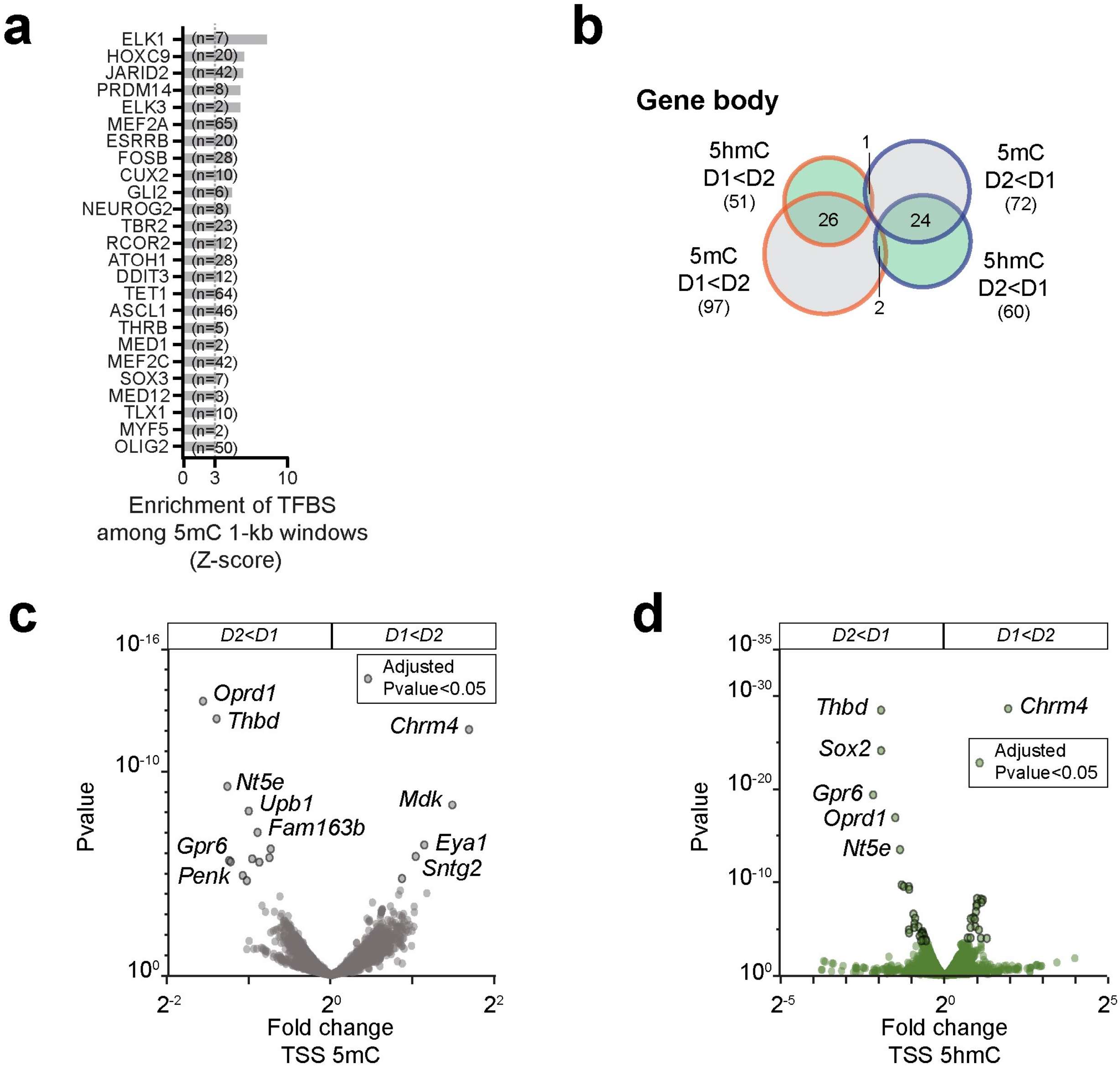

a Top 25 transcription factor binding sites enriched among the significant 1-kb methylation windows. **b** Venn diagram of the gene body 5mC and 5hmC differences, in D1 and D2 neurons. **c** Volcano plot of TSS 5mC differences between D1 and D2 neurons (TSS +/-2 kb). **d** Volcano plot of TSS 5hmC differences between D1 and D2 neurons (TSS +/-2 kb).

Supplementary Figure 5: Relationships between DNA modifications and gene expression.

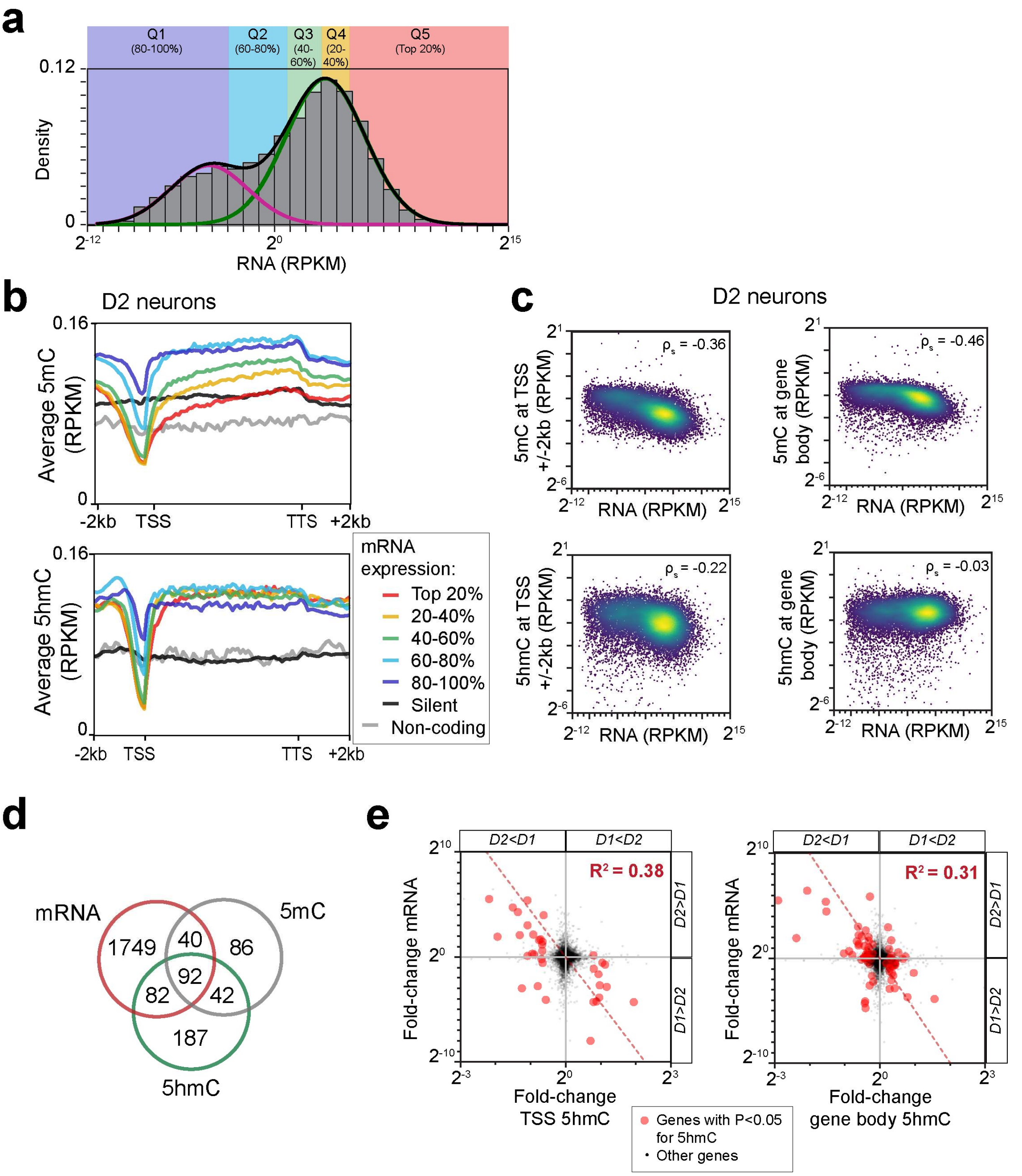

a Distribution of the mRNA expression, split into quintiles. The distribution of expression fits a bimodal Gaussian distribution (purple and green curves). **b** Metagene profiles of 5mC according to gene expression levels in D2 neurons. In these plots the gene body lengths are normalized while the preceding and following 2-kb are linear. **c** Density plots of expressed coding genes in D2 neurons, comparing the mRNA levels to the 5mC levels or 5hmC at TSS or gene body. $\rho_{s,}$, Spearman correlation coefficient. **d** Venn diagram of the significant differences between D1 and D2 neurons in mRNA, 5mC and 5hmC (TSS, gene body or 1-kb windows analyses). **e** Scatter plots of the D1/D2 differences in mRNA levels as a function of the changes in 5hmC at TSS and gene body.

Supplementary Figure 6: DNA modifications strand asymmetry.

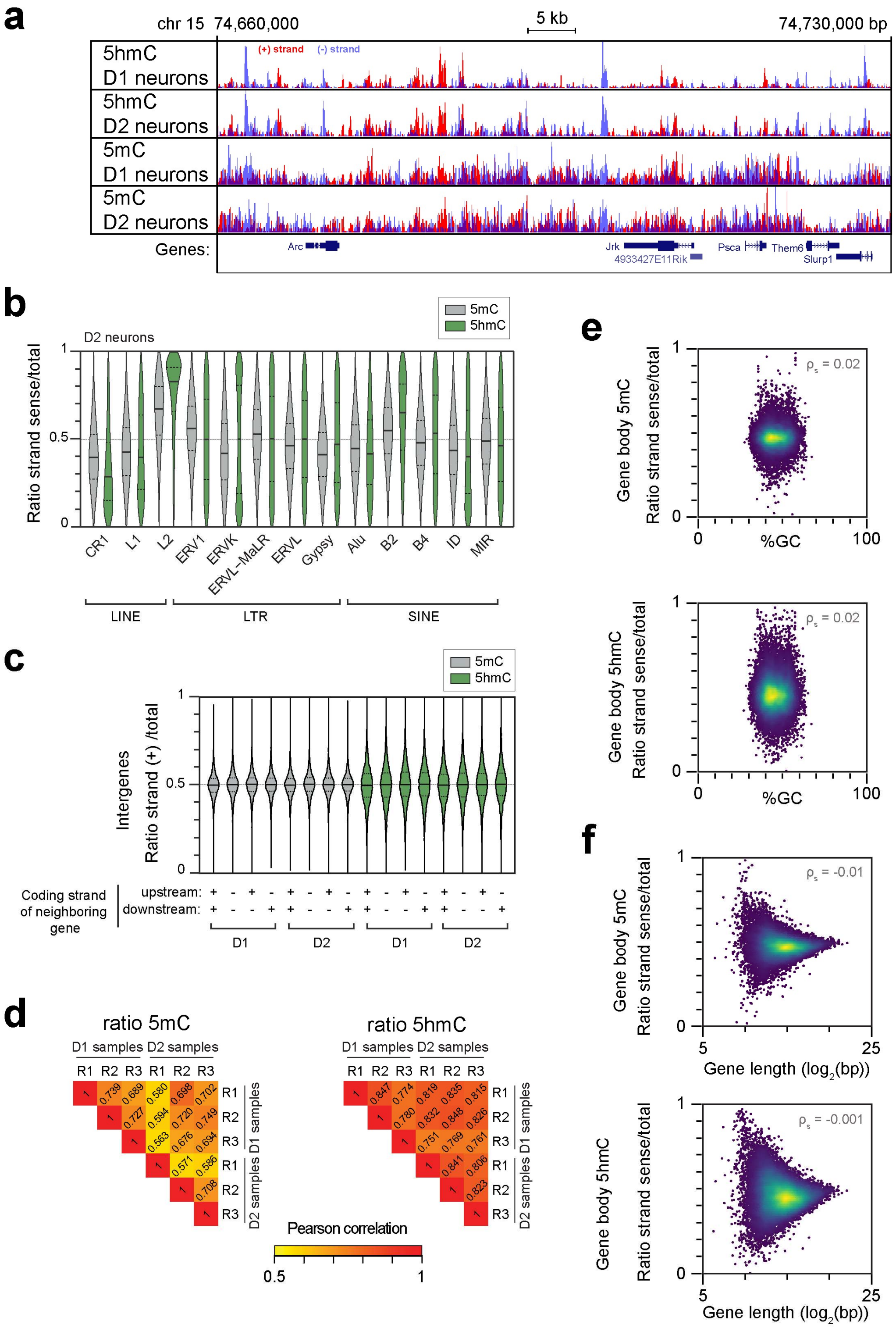

a Example of genome browser view of 5hmC asymmetry between both DNA strands compared to 5mC in D1 and D2 neurons. **b** Asymmetry of transposable elements in D2 neurons. **c** Strand asymmetry ratio in intergenic regions (<50 kb). **d** Correlation of 5hmC or 5mC asymmetry ratios on gene bodies between replicates. **e** Density plot of 5mC or 5hmC strand asymmetry in gene body as a function of GC content. ρ_s, Spearman correlation coefficient. **f** Density plot of 5mC or 5hmC of the strand asymmetry in gene body as a function of gene length. ρ_s, Spearman correlation coefficient.

Supplementary Figure 7: 5hmC strand asymmetry in coding genes increases with expression levels, and relation with CH strand asymmetry.

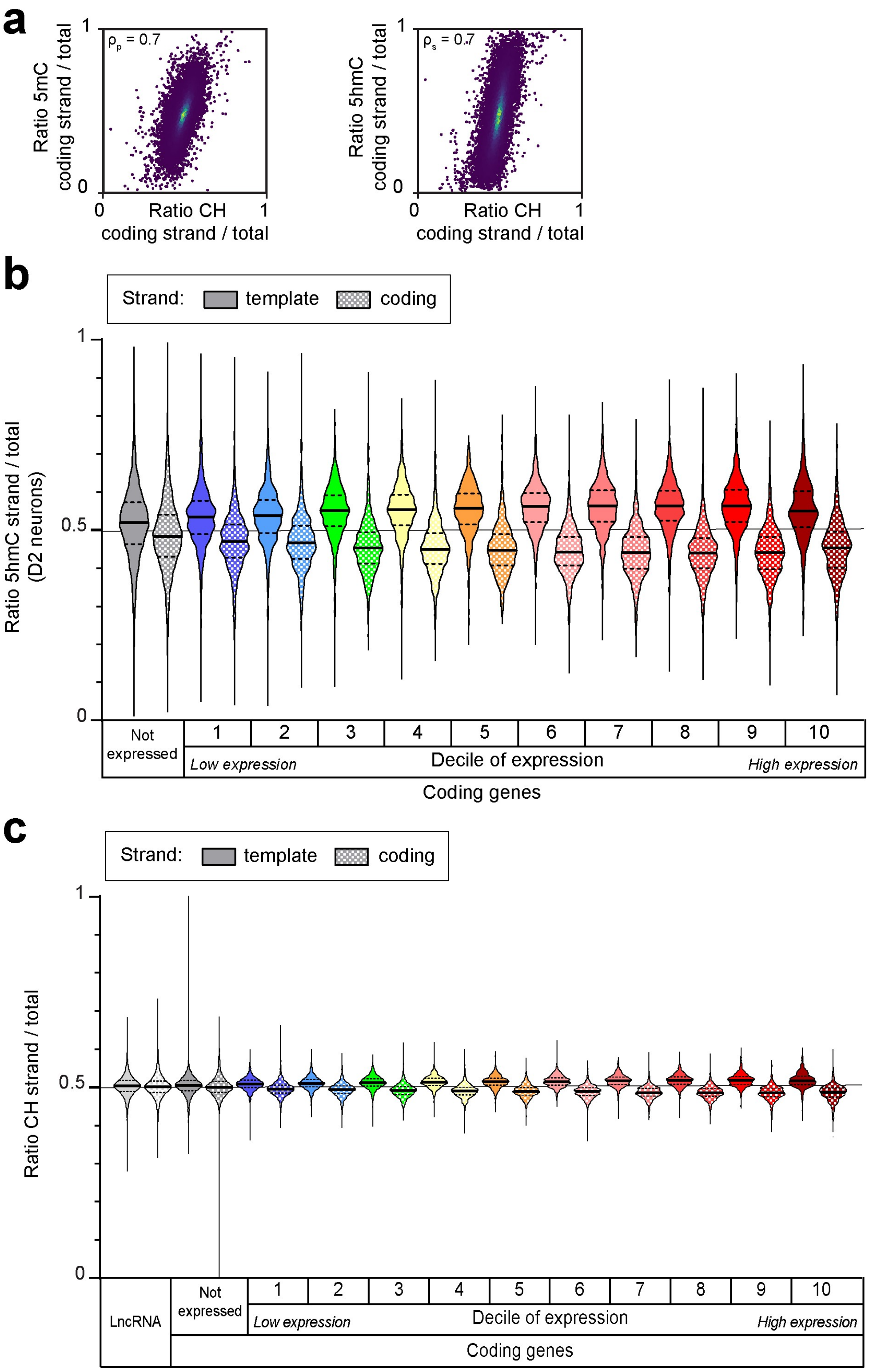

a Density plot of 5mC or 5hmC of the strand asymmetry in gene body as a function of the strand asymmetry in CH. ρ_s,, Spearman correlation coefficient. **b** Violin plots of the 5hmC asymmetry ratios in D2 neurons on template and coding strands of coding genes with different expression levels. **c** Violin plots of the CH asymmetry ratios on template and coding strands of lncRNAs and coding genes with different expression levels. In **b**,**c**, horizontal solid lines are medians and dotted lines quartiles.
