# Supplementary material for: DNA methylation and hydroxymethylation characterize the identity of D1 and D2 striatal projection neurons
Source: Commun Biol. 2022 Dec 1;5:1321. doi: 10.1038/s42003-022-04269-w (PMC9715678; doi:10.1038/s42003-022-04269-w)
Supplement: Supplementary file 2 — Description of Additional Supplementary Files [file 42003_2022_4269_MOESM2_ESM.docx]

**Description of Additional Supplementary Files**

File name:

Supplementary table 1

Description: Differentially expressed genes

Supplementary table 2

Description: List of differentially methylated 1-kb windows

Supplementary table 3

Description: List of differentially hydroxymethylated 1-kb windows

Supplementary table 4

Description: Clusterization of the differentially methylated 1-kb windows

Supplementary table 5

Description: List of the main methylation clusters

Supplementary table 6

Description: Clusterization of the differentially hydroxymethylated 1-kb windows

Supplementary table 7

Description: List of the main hydroxymethylation clusters

Supplementary table 8

Description: List of genes overlapping the differentially methylated 1-kb windows

Supplementary table 9

Description: List of genes overlapping the differentially hydroxymethylated 1-kb windows

Supplementary table 10

Description: Differentially methylated genes at their gene body

Supplementary table 11

Description: Differentially hydroxymethylated genes at their gene body

Supplementary table 12

Description: Differentially methylated genes at their TSS

Supplementary table 13

Description: Differentially hydroxymethylated genes at their TSS

Supplementary table 14

Description: Summary table of differential genes
