# Supplementary material for: DNA methylation and hydroxymethylation characterize the identity of D1 and D2 striatal projection neurons
Source: Commun Biol. 2022 Dec 1;5:1321. doi: 10.1038/s42003-022-04269-w (PMC9715678; doi:10.1038/s42003-022-04269-w)
Supplement: Supplementary file 4 — Reporting Summary [file 42003_2022_4269_MOESM4_ESM.pdf]

nature portfolio

Corresponding author(s): Jean-Antoine Girault, Lucile Marion-Poll

Last updated by author(s): Nov 5, 2022

Reporting Summary

Nature Portfolio wishes to improve the reproducibility of the work that we publish. This form provides structure for consistency and transparency in reporting. For further information on Nature Portfolio policies, see our Editorial Policies and the Editorial Policy Checklist.

Statistics

For all statistical analyses, confirm that the following items are present in the figure legend, table legend, main text, or Methods section.

n/a	Confirmed	
☐	☒	The exact sample size (*n*) for each experimental group/condition, given as a discrete number and unit of measurement
☐	☒	A statement on whether measurements were taken from distinct samples or whether the same sample was measured repeatedly
☐	☒	The statistical test(s) used AND whether they are one- or two-sided<br>*Only common tests should be described solely by name; describe more complex techniques in the Methods section.*
☒	☐	A description of all covariates tested
☐	☒	A description of any assumptions or corrections, such as tests of normality and adjustment for multiple comparisons
☐	☒	A full description of the statistical parameters including central tendency (e.g. means) or other basic estimates (e.g. regression coefficient) AND variation (e.g. standard deviation) or associated estimates of uncertainty (e.g. confidence intervals)
☐	☒	For null hypothesis testing, the test statistic (e.g. F, t, r) with confidence intervals, effect sizes, degrees of freedom and P value noted<br>*Give P values as exact values whenever suitable.*
☒	☐	For Bayesian analysis, information on the choice of priors and Markov chain Monte Carlo settings
☒	☐	For hierarchical and complex designs, identification of the appropriate level for tests and full reporting of outcomes
☐	☒	Estimates of effect sizes (e.g. Cohen's *d*, Pearson's *r*), indicating how they were calculated

Our web collection on statistics for biologists contains articles on many of the points above.

Software and code

Policy information about availability of computer code

Data collection	no software was used
Data analysis	FASTQC 0.10.1, FASTX-toolkit sofware suite 0.0.13, BWA 0.7.5, Tophat 2.0.10, Samtools 0.1.19, MEDIPS 1.16.0, Rsubread 1.28.0, DESeq2 1.27.32, Limma 3.36.5, Bedtools 2.29.2, Metagene 2.4.3, Rtracklayer v1.50.0, BSgenome.Mmusculus.UCSC.mm10 3.15, GenomicRanges v3.15, Bsgenome v1.58.0, GO Consortium online tool (http://geneontology.org/), Venny (https://www.stefanjol.nl/venny), IGV 2.0

For manuscripts utilizing custom algorithms or software that are central to the research but not yet described in published literature, software must be made available to editors and reviewers. We strongly encourage code deposition in a community repository (e.g. GitHub). See the Nature Portfolio guidelines for submitting code & software for further information.

Data

Policy information about availability of data

All manuscripts must include a data availability statement. This statement should provide the following information, where applicable:

- Accession codes, unique identifiers, or web links for publicly available datasets
- A description of any restrictions on data availability
- For clinical datasets or third party data, please ensure that the statement adheres to our policy

The raw sequencing data (FASTQ files) generated in this study have been deposited in the GEO database under accession code GSE186572

March 2021

Field-specific reporting

Please select the one below that is the best fit for your research. If you are not sure, read the appropriate sections before making your selection.

☒ Life sciences ☐ Behavioural & social sciences ☐ Ecological, evolutionary & environmental sciences

For a reference copy of the document with all sections, see nature.com/documents/nr-reporting-summary-flat.pdf

Life sciences study design

All studies must disclose on these points even when the disclosure is negative.

Sample size	no sample-size calculation was performed
Data exclusions	no data exclusion
Replication	All the data is presented
Randomization	Not relevant to the study, as we used two different mouse lines without treatment
Blinding	Not relevant to the study, as we used two different mouse lines without treatment

Reporting for specific materials, systems and methods

We require information from authors about some types of materials, experimental systems and methods used in many studies. Here, indicate whether each material, system or method listed is relevant to your study. If you are not sure if a list item applies to your research, read the appropriate section before selecting a response.

Materials & experimental systems

n/a	Involved in the study	
☐	☒	Antibodies
☒	☐	Eukaryotic cell lines
☒	☐	Palaeontology and archaeology
☐	☒	Animals and other organisms
☒	☐	Human research participants
☒	☐	Clinical data
☒	☐	Dual use research of concern

Methods

n/a	Involved in the study	
☒	☐	ChIP-seq
☐	☒	Flow cytometry
☒	☐	MRI-based neuroimaging

Antibodies

Antibodies used	monoclonal anti-GFP antibodies clones 19F7 and 19C8 (Memorial Sloan-Kettering Monoclonal Antibody Facility, New York), 5-methylcytosine monoclonal mouse antibody clone 33D3 (Mab-081, Diagenode), 5-hydroxymethylcytosine monoclonal mouse antibody (Mab-31HMC, Diagenode)
Validation	Antibodies from Diagenode have been validated by the manufacturer (see website), and we used their DNA control package to optimize the conditions of the IP. The GFP antibodies have been described in the original Nature Protocols article PMID 24810037.

Animals and other organisms

Policy information about studies involving animals; ARRIVE guidelines recommended for reporting animal research

Laboratory animals	male and female mice, 2-3 month old, C57Bl/6J background, Drd1a-EGFP-L10a or Drd2-EGFP-L10a mutants
Wild animals	the study did not involve wild animals
Field-collected samples	the study did not involve samples collected from the field
Ethics oversight	All the experiments were in accordance with the National Institutes of Health Guide for the Care and Use of Laboratory Animals and approved by Rockefeller University's Institutional Animal Care and Use Committee

Note that full information on the approval of the study protocol must also be provided in the manuscript.

Flow Cytometry

Plots

Confirm that:

☒ The axis labels state the marker and fluorochrome used (e.g. CD4-FITC).

☒ The axis scales are clearly visible. Include numbers along axes only for bottom left plot of group (a 'group' is an analysis of identical markers).

☒ All plots are contour plots with outliers or pseudocolor plots.

☒ A numerical value for number of cells or percentage (with statistics) is provided.

Methodology

Sample preparation	Nuclei from mouse striata were dissociated with a Dounce homogenizer, and purified by density using iodixanol
Instrument	BD FacsAriaII
Software	BD FACSDiva
Cell population abundance	More than 100,000 nuclei per sample were collected, with >97% purity (determined by flow cytometry of the post-sort fraction)
Gating strategy	Nuclei were gated by two criteria: the signal from DyeCycle Ruby corresponding to single nuclei and a GFP signal above background fluorescence (as assessed by comparison with nuclei from a wild-type littermate mouse)

☒ Tick this box to confirm that a figure exemplifying the gating strategy is provided in the Supplementary Information.
